# Temporal changes in tongue color during immune checkpoint inhibitor therapy in patients with non-small-cell lung cancer: a prospective observational study using digital tongue diagnosis

**DOI:** 10.3389/or.2025.1697252

**Published:** 2025-12-09

**Authors:** Eunbyul Cho, Woosu Choi, Jun Hyeok Lim, Ji Woong Son, Seung Hun Jang, Seung Hyeun Lee, Jong Gwon Choi, In-Jae Oh, Tae-Won Jang, Seong Hoon Yoon, Seung Joon Kim, Chang-Min Choi, Sung Yong Lee, Mi Mi Ko, Mi-Kyung Jeong

**Affiliations:** 1 Department of Diagnostics, College of Korean Medicine, Wonkwang University, Iksan, Republic of Korea; 2 Digital Health Research Division, Korea Institute of Oriental Medicine, Daejeon, Republic of Korea; 3 Division of Pulmonology, Department of Internal Medicine, Inha University Hospital, Inha University College of Medicine, Incheon, Republic of Korea; 4 Division of Pulmonology, Department of Internal Medicine, Konyang University Hospital, Daejeon, Republic of Korea; 5 Division of Pulmonary, Allergy, and Critical Care Medicine, Department of Internal Medicine, Hallym University Sacred Heart Hospital, Hallym University College of Medicine, Anyang, Gyeonggi, Republic of Korea; 6 Division of Pulmonary and Critical Care Medicine, Department of Internal Medicine, Kyung Hee University Hospital, Kyung Hee University College of Medicine, Seoul, Republic of Korea; 7 Department of Oncology- Hematology, Konyang University Hospital, Daejeon, Republic of Korea; 8 Department of Internal Medicine, Chonnam National University Medical School and Hwasun Hospital, Jeonnam, Republic of Korea; 9 Department of Internal Medicine, Kosin University Gospel Hospital, Busan, Republic of Korea; 10 Department of Internal Medicine, Pusan National University Yangsan Hospital, Yangsan, Republic of Korea; 11 Department of Internal Medicine, Seoul St. Mary’s Hospital, Postech-Catholic University Biomedical Engineering Institute, College of Medicine, The Catholic University of Korea, Seoul, Republic of Korea; 12 Department of Pulmonary and Critical Care Medicine, Department of Oncology, Asan Medical Center, University of Ulsan College of Medicine, Seoul, Republic of Korea; 13 Division of Pulmonary, Allergy, and Critical Care Medicine, Korea University Guro Hospital, Seoul, Republic of Korea; 14 KM Science Research Division, Korea Institute of Oriental Medicine, Daejeon, Republic of Korea; 15 KM Convergence Research Division, Korea Institute of Oriental Medicine, Daejeon, Republic of Korea

**Keywords:** lung cancer, tongue diagnosis, digital tongue image, immunotherapy, prognosis

## Abstract

**Background:**

Tongue diagnosis (TD), a key component of traditional East Asian medicine, employs a unique pattern-based diagnostic system. Digital TD enables quantitative assessment of tongue characteristics, like body and coating color, enhancing objectivity and reproducibility. While abnormal tongue features (including dark red, bluish, or pale appearance) have been documented in patients with cancer, the relationship between longitudinal changes in tongue characteristics and immune checkpoint inhibitor (ICI) treatment response or survival outcomes in non-small-cell lung cancer (NSCLC) remains underexplored. This multicenter, prospective, observational study investigated whether longitudinal tongue changes differ by ICI response and predict survival in patients with NSCLC.

**Methods:**

We enrolled patients with stage IIIB, IIIC, or IV NSCLC scheduled to receive second-line or subsequent pembrolizumab or atezolizumab following first-line platinum-based therapy failure. Digital tongue images were collected every 9 weeks from baseline to week 45. Linear mixed models evaluated temporal parameter changes and compared responders (durable clinical benefits ≥6 months) *versus* nonresponders. Multivariate Cox models adjusted for sex and age assessed tongue lightness changes as a prognostic value for progression-free survival (PFS) and overall survival (OS). Survival distributions were compared using Kaplan–Meier curves.

**Results:**

Of 170 enrolled participants, 140 were included in the analysis. Early in treatment, tongue lightness decreased in the body, fur, root, and center areas in both responders and nonresponders; however, the darkening was more pronounced in nonresponders, with significant visit-by-response interaction effects. In multivariate Cox analysis, lightness changes of the tongue body were significantly associated with PFS (hazard ratio [HR] = 0.93; 95% confidence interval [CI], 0.88–0.99; *p* = 0.019) and showed a trend for OS (HR = 0.93; 95% CI, 0.86–1.00; *p* = 0.062). Lightness changes of the tongue center were also significantly associated with PFS (HR = 0.95; 95% CI, 0.90–0.99; *p* = 0.027). Kaplan–Meier analysis confirmed that patients with a greater decrease in tongue body lightness had significantly shorter OS (*p* = 0.049).

**Conclusion:**

Digital TD diagnosis, particularly monitoring tongue lightness changes, may provide a valuable noninvasive prognostic tool for patients with NSCLC undergoing ICI therapy. It offers information for both PFS and OS, potentially complementing current biomarkers for cancer immunotherapy.

## 1 Introduction

Immune checkpoint inhibitors (ICIs) have revolutionized cancer treatment over the past decade, showing remarkable clinical benefits (1). Despite their promising outcomes, the clinical utility of ICIs is often limited by resistance and short-lived responses in some patients (2). Non-small-cell lung cancer (NSCLC) represents one of the major indications for ICI therapy, with a prevalence of 40.9 cases per 100,000 in the United States as of 2017 (3), maintaining relatively high incidence rates among cancer types (4). Treatment responses to ICIs in patients with NSCLC widely vary, ranging from prolonged survival to lack of response and immune-related adverse events (5). Although programmed death-ligand 1 (PD-L1) expression serves as an approved predictive biomarker for ICI therapy in patients with NSCLC, its measurement accuracy and prognostic power remain limited (6).

Several clinical findings are useful prognostic and predictive factors for ICI treatment efficacy in NSCLC, including sex, body mass index, overweight and obesity status, Eastern Cooperative Oncology Group Performance Status (ECOG-PS), baseline corticosteroid or antibiotic use, and liver metastases (6,7). However, these clinical parameters often show inconsistent results across different studies and patient populations, limiting their universal applicability. Additionally, blood parameters, such as C-reactive protein, baseline lactate dehydrogenase levels, and neutrophil-to-lymphocyte ratio, have shown promise, but precise cutoff values remain unclear and require invasive blood sampling procedures. Given the current insufficiency of reliable prognostic factors for survival prediction in patients with NSCLC receiving ICI therapy (8), the clinical relevance of noninvasive, easily accessible diagnostic indicators is worth exploring.

Tongue diagnosis (TD), an important objective sign used for pattern identification (PI) in traditional East Asian medicine (TEAM), is used to identify physiological functions and pathological changes in the human body (9). In TEAM, TD features indicate disease severity, internal organ condition, and metabolic status expressed as “cold and heat” and “excess and deficiency” (10). TD is primarily performed by Korean medicine doctors (KMDs) with the naked eye in real-world settings (9); however, a digital TD system has been developed to meet the growing demand for accuracy and reproducibility. This system has enabled the measurement of physical indicators, such as tongue color, shape, depth, and thickness, using three-dimensional images (11). Its diagnostic results have shown high reliability (Cohen’s κ 0.84–1.00), exceeding the agreement between the system and KMDs, as well as among KMDs themselves (12,13).

Using the digital TD system, several characteristic features have been identified in patients with breast, esophageal, liver, lung, stomach, rectal, and ovarian cancers, demonstrating the clinical significance of observing the changes in the tongue characteristics in these patients (14). Previous studies have reported specific tongue characteristics in patients with cancer, including dark red (15,16) or blue (17,18) color, paleness of the tongue (19), and thick or yellowish coating (19,20). Moreover, the tongue tends to be thicker (16,21) or have a larger area (22) in patients with cancer than in healthy individuals. Although previous studies have used cameras or a digital TD system for tongue characterization, they primarily focused on comparing tongue data between cancer stages or between healthy individuals and patients with cancer, rather than analyzing posttreatment changes or treatment response associations (14).

Lung cancer represents a major cancer type with high incidence and mortality rates, making the development of effective prognostic factors particularly crucial (23,24). Therefore, this study aimed to analyze longitudinal changes in tongue characteristics during ICI treatment in patients with NSCLC using digital TD and to examine the differences in these parameters between responders and nonresponders. We evaluated whether changes in tongue variables over time can distinguish treatment response patterns and potentially serve as objective indicators for monitoring treatment effects in patients with advanced NSCLC receiving immunotherapy.

## 2 Materials and methods

### 2.1 Participants

This multicenter, prospective, observational study included patients aged ≥19 years diagnosed with stage IIIB, IIIC, or IV NSCLC and scheduled to receive the ICIs pembrolizumab or atezolizumab alone as a second-line treatment or subsequent therapy after the failure of first-line platinum-based therapy. Patients with a survival expectancy of ≥6 months, an ECOG-PS score ≤2, and who volunteered to participate after receiving a detailed explanation of the study were enrolled. After this study received institutional review board approval, the patients were recruited from 10 university hospitals in South Korea. Screening and data collection were performed according to a previously reported protocol (25).

### 2.2 Digital tongue diagnosis

The patients were instructed to refrain from engaging in all oral activities, including eating, brushing, and gargling, 2 h prior to testing. To ensure consistent tongue positioning and reduce inter-individual variability in tongue shape and size, real-time grid guidelines were provided to subjects during image acquisition to help them adjust their posture (26). TD was performed six times from baseline to week 45 at intervals of 9 weeks. The patients were followed up until withdrawal, death, or study completion. Digital TD equipment (computerized tongue image analysis system (TAS); Korea Institute of Oriental Medicine, Daejeon, Korea) was used to acquire images of the tongue and evaluate the colors of the body of the tongue and coating, amount of coating, and toothmarks within the examined area. The equipment used in this study has an algorithm to increase repeatability and diagnostic accuracy using indirect illumination and feedback gridlines (27,28). Moreover, it was designed to meet the international standards ISO 20498-1, 20498-2, 20498-3, 20498-4, and 20498-5. Physical quantities related to TD were automatically acquired. Data that could not be analyzed owing to poor posture, tongue staining, or breathing on the lens were excluded.

**FIGURE 1:**
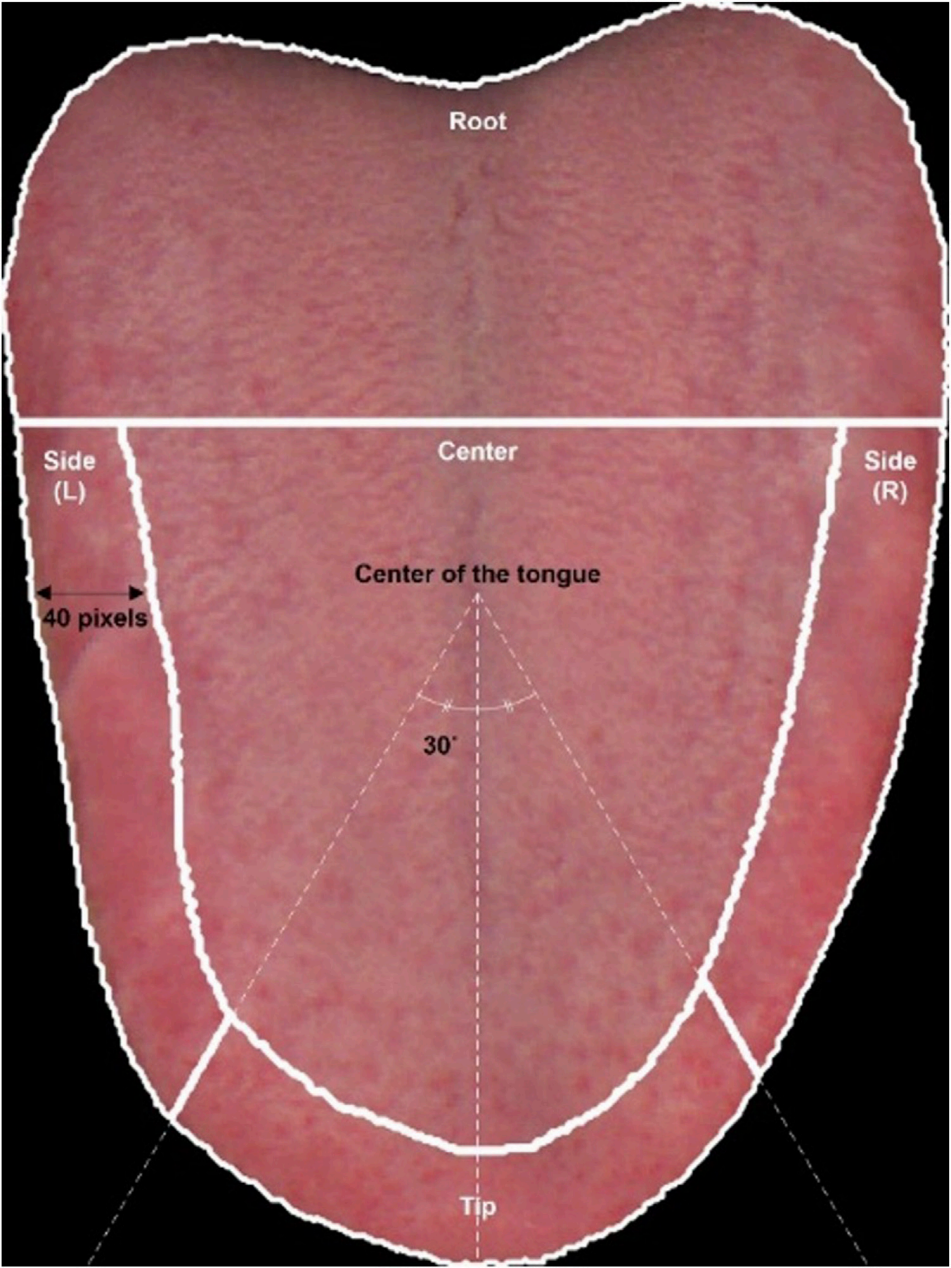
Division of the area of the tongue into the root, center, side, and tip. The root is the anterior one-third of the tongue, including the beginning of the tongue. The center is the area >40 pixels from the edge of the tongue. On the edge, which is the area within 40 pixels from the rim of the tongue, the area within 30° to the left and right of the center of the tongue is the tip. The remaining areas are the sides. L, left; R, right.

The tongue was divided into the root, center, tip, and sides to analyze tongue color in each region. The length of the tongue was divided into thirds from the root to the tip of the tongue. The area where the tongue begins was defined as the root; the area including a distance of 40 pixels from the edge of the tongue to the central point of the tongue, excluding the root, was defined as the edge; and the remaining area, excluding the root and edge, was defined as the center. The portion of the edge where the angle between the center and tip of the tongue was within the range of ±30° from the point connecting the center of the tongue to the tip was defined as the tip. The remaining areas were defined as the side (Figure 1).

Pixel values in the tongue region were converted from the red-green-blue color space to the Commission Internationale del’Éclairage (CIE) L*a*b* color space, which is similar to human color perception (29). The CIE L*, CIE a*, and CIE b* values represent the lightness, saturation of red and green, and saturation of blue and yellow, respectively. The redness shows a proportional increase with the CIE a* color value, whereas it shows an inverse relationship with the CIE b* color value (29,30). The color of the tongue coating on the entire tongue was expressed as L, a, and b of fur (tongue coating) according to the CIE classification system. To ensure inter-site consistency and reproducibility, ColorChecker Passport calibration was implemented within the TD device during imaging for rigorous quality control. Color correction analysis using CIEDE2000 demonstrated reliable device calibration across centers with mean ΔE_00_ values below established perceptibility thresholds (31,32). Based on a previous study that reported a proportional relationship between the area and thickness of the tongue coating (33), the ratio of the tongue coating to the total tongue area was used to objectively measure the thickness of the tongue coating in the present study. Data that objectively quantified the number of toothmarks by expressing the curvature of the tongue edge as a frequency component were used in the present study (34).

### 2.3 Statistical analyses

The color of the body of the tongue was divided into three categories—red, light pink, and pale—using the tongue color analysis algorithm developed by the Korea Institute of Oriental Medicine. Based on a previous study (35) that suggested cutoff values for tongue coating levels for the same digital TD device used in the present study, tongue coating ratios were categorized as follows: no coating (≤16.3782%), thin coating (16.3782< ≤ 28.527%), and thick coating (>28.527%) (11).

Statistical analyses were performed using R software (version 4.4.2; R Core Team). Linear mixed models were used to evaluate changes in TD variables over time during ICI therapy. Each tongue parameter was set as the dependent variable, with “visit” as a fixed effect and patient ID as a random effect. Random effects were specified as random intercepts by patient ID, without random slopes, to preserve model stability given the limited number of repeated measures per patient. This model accounts for within-subject correlation in repeatedly measured data while assessing temporal changes. Post-hoc testing for differences between baseline (Visit 1) and each subsequent visit was performed using Tukey’s method to adjust for multiple comparisons.

To analyze differences in temporal changes of tongue parameters between responders (patients with durable clinical benefits, defined as complete or partial response and stable disease lasting >6 months) (36) and nonresponders to ICI therapy, linear mixed models, including interaction terms, were used. For each tongue parameter, fixed effects included visit, response status, and interaction, with patient ID as a random intercept. Sex and age were included as covariates. Models were constructed with a random intercept structure (no random slopes). Analyses were restricted to the early treatment phase (Visits 1–3). When the interaction term was statistically significant, post-hoc testing was performed to assess differences between Visit 1 and subsequent visits within each group.

To assess the prognostic value of lightness-related tongue changes (Δ values calculated as the difference between the last available visit and baseline), multivariate Cox proportional hazards models were used for progression-free survival (PFS) and overall survival (OS), adjusting for sex and age, which were significant prognostic factors in patients with NSCLC receiving ICI therapy in previous studies (37,38). Kaplan–Meier survival curves were plotted using the median Δ value as the cutoff to define high and low groups, and log-rank tests were used to compare survival distributions. A two-sided p-value of <0.05 was considered statistically significant.

## 3 Results

### 3.1 Basic characteristics of the participants

Of the total 170 participants, 140 were included in the analysis, excluding 17 who withdrew consent and 13 with missing data for TD. The median age of the included patients was 68.5 (range, 42–87) years. Baseline demographic characteristics, including sex, age, history of smoking, tumor–node–metastasis stage, ECOG-PS score, histological type, and epidermal growth factor receptor at the baseline, showed no significant difference between the responder and nonresponder groups. Types of ICI and PD-L1 expression were associated with treatment response to ICIs (Table 1).

**TABLE 1:** Baseline characteristics of the participants.

Characteristics	Total (n = 140)	Responder group (n = 44)		Nonresponder group (n = 96)		*p*-value
		n	%	n	%	
Sex						
Male	115	33	28.7	82	71.3	0.157
Female	25	11	44	14	56	
Age						
≥65	96	30	31.3	66	68.8	>0.999
<65	44	14	31.8	30	68.2	
History of smoking						
Current smoker	18	6	33.3	12	66.7	0.815
Former smoker	97	29	29.9	68	70.1	
Never smoked	25	9	36.0	16	64.0	
TMN stage						
III	13	6	46.2	7	53.8	0.218
IVA	69	24	34.8	45	65.2	
IVB	58	14	24.1	44	75.9	
ECOG						
0	13	3	23.1	10	76.9	0.765
1	110	35	31.8	75	68.2	
2	17	6	35.3	11	64.7	
ICIs						
Atezolizumab	94	20	21.3	74	78.7	<0.001*
Pembrolizumab	46	24	52.2	22	47.8	
Histological type						
Non-squamous	76	28	36.8	48	63.2	0.147
Squamous	64	16	25.0	48	75.0	
PD-L1 expression^a^						
Negative	40	7	17.5	33	82.5	<0.001*
<50	53	12	22.6	41	77.4	
≥50	46	25	54.3	21	45.7	
EGFR mutation						
Negative	128	41	32.0	87	68.0	0.753
Positive	12	3	25	9	75	

^a^(n=1).

The TD algorithm classified the tongue color as pale, light pink, and red for 55 (39.3%), 54 (38.6%), and 31 (22.1%) patients, respectively. Based on the tongue coating ratio, 26 (18.6%), 83 (59.3%), and 31 (22.1%) patients had no coating, thin coating, and thick coating, respectively (Table 2).

**TABLE 2:** Tongue color and tongue coating ratio at baseline.

Tongue color	Tongue coating			
	No coating	Thin coating	Thick coating	Total
Pale	7 (5%)	25 (17.9%)	23 (16.4%)	55 (39.3%)
Light pink	5 (3.6%)	42 (30%)	7 (5%)	54 (38.6%)
Red	14 (10%)	16 (11.4%)	1 (0.7%)	31 (22.1%)
Total	26 (18.6%)	83 (59.3%)	31 (22.1%)	140 (100%)

### 3.2 Changes in tongue diagnosis variables over time

**FIGURE 2:**
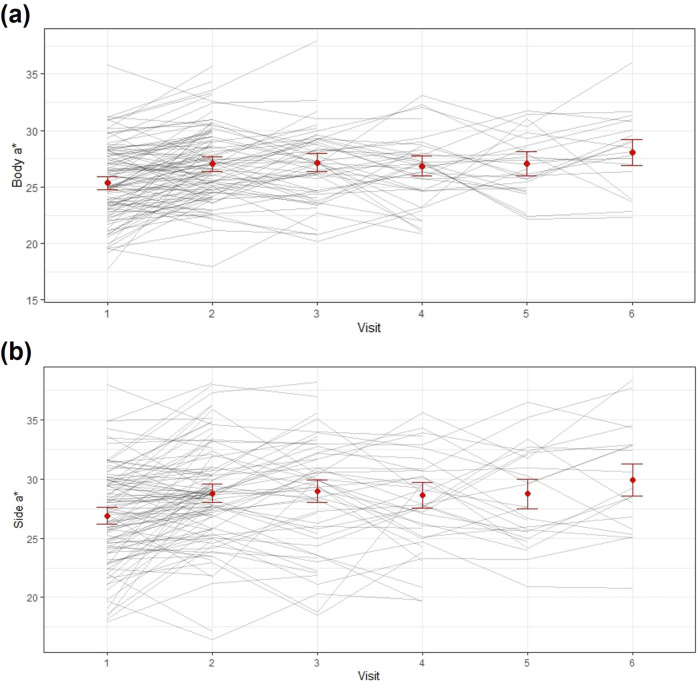
Individual and mean changes in CIE a* values of the tongue. **(a)** Body and **(b)** side regions.

Analysis of longitudinal changes of all included participants (n = 140) during ICI therapy showed significant changes in multiple variables compared with the baseline (Visit 1). CIE L* values significantly decreased at Visit 2 in the body (mean difference [MD] = −1.13, *p* = 0.001), fur (MD = −2.99, *p* < 0.001), and center (MD = −1.60, *p* < 0.001) areas. CIE a* values significantly increased across all regions (body, fur, root, center, side, and tip) (*p* < 0.05, respectively) at Visit 2, with body and side areas maintaining consistently elevated values throughout Visits 2–6 (Figure 2; Table 3; Supplementary Additional File S1, S2). CIE b* values showed no significant changes from baseline in any area throughout the observation period.

**TABLE 3:** Summary of change over time for tongue variables that were significantly different from baseline.

Variable	Baseline mean (SE)	Visit	Changes from baseline (estimate)	Time effect (*p*-value)
Body L*	53.6 (0.278)	2	−1.13	0.001
Fur L*	51.1 (0.589)	2	−2.99	<0.001
Center L*	56.6 (0.340)	2	−1.60	<0.001
Body a*	25.3 (0.291)	2	1.70	<0.001
		3	1.81	<0.001
		4	1.52	0.009
		5	1.72	0.013
		6	2.72	<0.001
Fur a*	13.0 (0.233)	2	0.93	0.012
Root a*	18.6 (0.308)	2	1.60	<0.001
		3	1.72	<0.001
Center a*	23.0 (0.351)	2	1.99	<0.001
		3	1.93	0.001
		6	2.81	0.002
Side a*	26.9 (0.357)	2	1.88	<0.001
		3	2.07	<0.001
		4	1.74	0.011
		5	1.84	0.030
		6	3.02	<0.001
Tip a*	28.6 (0.399)	2	2.18	<0.001
		3	2.43	<0.001
		6	3.20	0.001

The symbol * is an integral part of the variable names in the CIE 1976 color system.

### 3.3 Differences in changes in the tongue characteristics in response to immune checkpoint inhibitors

**FIGURE 3:**
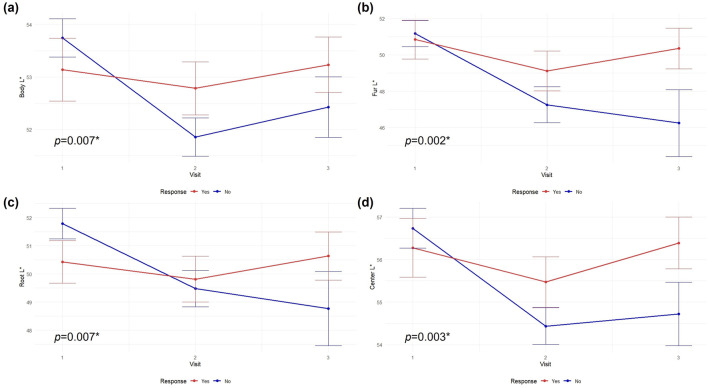
Changes in CIE L* values of the tongue. **(a)** Body, **(b)** fur, **(c)** root, and **(d)** center regions of the response and nonresponse groups.

Among the lightness-related parameters, body L, fur L, root L, and center L in the responder group showed a significant decrease over time (body L: estimate = −0.91, *p* < 0.001; fur L: estimate = −3.21, *p* < 0.001; root L: estimate = −1.44, *p* < 0.001; center L: estimate = −1.33, *p* < 0.001). The interaction terms (visit × response) for these variables were also significant (body L: estimate = 1.01, *p* = 0.007; fur L: estimate = 2.90, *p* = 0.002; root L: estimate = 1.63, *p* = 0.007; center L: estimate = 1.38, *p* = 0.003), indicating that tongue lightness in the body, fur, root, and center regions decreased more markedly over time in nonresponders than in responders. Side L and tip L showed significant within-group decreases over time in the responder group (side L: estimate = −0.75, *p* = 0.016; tip L: estimate = −0.68, *p* = 0.045), without significant interaction effects (Figure 3).

Redness-related variables, including body a (estimate = 1.31, *p* < 0.001), root a (estimate = 1.26, *p* < 0.001), center a (estimate = 1.58, *p* < 0.001), side a (estimate = 1.48, *p* < 0.001), and tip a (estimate = 1.70, *p* < 0.001), exhibited significant temporal changes, although interaction effects were not significant. Among the CIE b* parameters, fur b showed a significant decrease over time (estimate = −0.73, *p* = 0.012), whereas other b* values did not demonstrate significant temporal or interaction effects (Table 4; Supplementary Additional File S3).

**TABLE 4:** Comparison of tongue variable changes between responders and nonresponders during early ICI treatment (Visits 1–3).

Variable	Visit × response estimate	Standard error	t-value	p-value
Body L	1.01	0.37	2.76	0.007*
Body a	−0.40	0.35	−1.13	0.264
Body b	−0.67	0.28	−1.24	0.220
Fur L	2.90	0.92	3.15	0.002*
Fur a	0.17	0.35	0.49	0.623
Fur b	0.26	0.42	0.61	0.545
Root L	1.63	0.59	2.78	0.007*
Root a	−0.49	0.39	−1.26	0.211
Root b	−0.08	0.42	−0.20	0.842
Center L	1.38	0.44	3.12	0.003*
Center a	−0.67	0.46	−1.46	0.148
Center b	−0.29	0.33	−0.87	0.389
Side L	0.85	0.44	1.92	0.058
Side a	−0.41	0.42	−0.97	0.333
Side b	−0.17	0.25	−0.69	0.490
Tip L	0.67	0.48	1.38	0.171
Tip a	−0.45	0.52	−0.88	0.384
Tip b	−0.10	0.29	−0.35	0.727

Visit × response denotes the interaction effect, capturing whether the pattern of change over time differs between responders and nonresponders. *p < 0.05.

### 3.4 Association between tongue lightness changes and survival outcomes

In the multivariate Cox proportional hazards model adjusting for sex and age (Model1), ΔBodyL and ΔCenterL were significantly associated with PFS (ΔBodyL: hazard ratio [HR] = 0.93, 95% CI: 0.88–0.99, *p* = 0.019; ΔCenterL: HR = 0.95, 95% CI: 0.90–0.99, *p* = 0.027), suggesting that greater increases in tongue lightness in body and center areas were associated with lower risk of progression. Changes in fur and root region lightness (ΔFurL and ΔRootL) were not significantly associated with PFS. ΔFurL was significantly associated with OS, with greater increases in fur region lightness predicting improved survival outcomes (HR = 0.97; 95% CI, 0.94–1.00; *p* = 0.049). ΔBodyL showed a trend toward significance (HR = 0.93; 95% CI, 0.86–1.00; *p* = 0.062). ΔCenterL also showed borderline significance for OS (HR = 0.94; 95% CI, 0.89–1.00; *p* = 0.054) (Table 5). Kaplan–Meier survival analysis using the median value as the cutoff point demonstrated that patients with high ΔBodyL (≥median) had significantly shorter OS than those with low ΔBodyL (<median) (*p* = 0.049). In contrast, no significant differences in OS were observed for ΔFurL, ΔRootL, or ΔCenterL. Moreover, none of the lightness changes showed significant associations with PFS (all *p* > 0.05) (Supplementary Additional File S4).

**TABLE 5:** Associations of changes in tongue lightness parameters with PFS and OS.

Variable	PFS			OS		
	HR	95% CI	*p*-value	HR	95% CI	*p*-value
Model 1 (adjusting for sex and age)						
ΔBodyL	0.93	0.88–0.99	0.019*	0.93	0.86–1.00	0.062
ΔFurL	0.98	0.96–1.00	0.0880	0.97	0.94–1.00	0.049*
ΔRootL	0.97	0.94–1.01	0.1635	0.96	0.91–1.01	0.109
ΔCenterL	0.95	0.90–0.99	0.027*	0.94	0.89–1.00	0.054
Model 2 (adjusting for sex, age, PD-L1 expression, and ICI type)						
ΔBodyL	0.94	0.88–1.01	0.081	0.94	0.87–1.02	0.127
ΔFurL	0.98	0.95–1.01	0.160	0.97	0.94–1.00	0.089
ΔRootL	0.99	0.95–1.03	0.712	0.96	0.92–1.02	0.169
ΔCenterL	0.96	0.91–1.02	0.164	0.95	0.89–1.02	0.155

Hazard ratios (HRs) were calculated using multivariable Cox proportional hazards models adjusted for sex and age. ΔVariable indicates the change from Visit 1 to the last available visit. PFS, progression-free survival; OS, overall survival; HR, hazard ratio; CI, confidence interval; ICI, immune checkpoint inhibitor. HR < 1 indicates a protective effect (lower risk of the event with increased levels of the variable). *p < 0.05.

None of the tongue lightness parameters showed statistically significant associations with either PFS or OS in the multivariate Cox proportional hazards model (Model 2), after additional adjustment for PD-L1 expression and ICI type, which were associated with treatment response at baseline. ΔBodyL showed a trend toward significance for PFS (HR = 0.94; 95% CI, 0.88–1.01; p = 0.081). ΔFurL showed a trend for improved OS (HR = 0.97; 95% CI, 0.94–1.00; p = 0.089) (Table 5). Lightness changes did not show significant associations with PFS and OS in the Kaplan–Meier survival analysis (Supplementary Additional File S4).

## 4 Discussion

This study aimed to determine the changes in the tongue characteristics over time in patients with advanced NSCLC receiving pembrolizumab or atezolizumab. To minimize potential confounding factors affecting tongue appearance, we implemented standard protocols, including restrictions on food and beverage consumption prior to imaging, and ensured tongue images were acquired in an unstained state. Most patients had a normal tongue, a light pink-colored body of the tongue, and a thin coating on the tongue at baseline. We identified significant longitudinal changes in tongue color variables, particularly in lightness (CIE L*) and redness (CIE a*) parameters. Notably, early-phase differences in lightness-related changes were observed between responders and nonresponders, with nonresponders showing significantly greater decreases in tongue lightness (darker tongues) over time in body, fur, root, and center regions than responders. The tongue generally became redder over time, without significant differences between responders and nonresponders.

Multivariate Cox proportional hazards analysis revealed that increases in body and center lightness were associated with longer PFS and that increases in fur lightness were associated with longer OS. In the Kaplan–Meier survival analysis using the median value as the cutoff, most results were not statistically significant. Our findings suggest that digital TD monitoring, particularly focusing on tongue lightness changes, may serve as a valuable prognostic tool during ICI therapy in patients with NSCLC, addressing the current limitations of biomarkers in cancer immunotherapy.

Previous cross-sectional studies have reported the association between tongue lightness and cancer, although results have been inconsistent (14,19,39). These discrepancies may stem from differences in study design, cancer types, and measurement methodologies. Although studies in this area remain limited and findings are not yet consistent, studies continue to explore tongue body and coating color as potential screening tools for cancer presence and progression, often in conjunction with other indicators, such as blood parameters and microbiome analysis (19,39). In our study, ΔBodyL demonstrated significant associations with both PFS and borderline significance with OS, suggesting that longitudinal changes in tongue lightness during treatment may provide prognostic information beyond baseline assessments. Given the current lack of reliable prognostic biomarkers for ICI therapy, our findings suggest the potential utility of tongue lightness changes as a noninvasive prognostic indicator for patients with NSCLC receiving immunotherapy, warranting further investigation in larger cohorts.

PI is a core diagnostic framework in traditional medicine and is officially recognized in the International Classification of Diseases (ICD)-11 (40). Among various diagnostic tools, TD has long served as a key visual indicator for PI and remains widely used in TEAM (41–43). In recent years, efforts have been made to incorporate objective data from diagnostic instruments to address the subjectivity of traditional assessments (9,44,45). However, internationally comparable and quantitative PI data remain limited (46). This prospective study contributes to filling this gap by providing objective digital TD data and statistical analysis in patients with NSCLC receiving ICI therapy.

The tongue is considered a valuable prognostic indicator in traditional Korean medicine, as it reflects the condition of internal organs and disease severity (9). Accordingly, this study explored whether tongue characteristics in patients with NSCLC can serve as predictive markers of treatment response. Overall, patients exhibited darker and redder tongues at Visit 2 than at baseline, as evidenced by decreased CIE L* values in the body, fur, and center areas and increased CIE a* values across all regions. Longitudinal analysis revealed distinct changes in lightness (L*) and redness (a*) values during early treatment. Significant interaction effects in the body, fur, root, and center regions indicated that nonresponders experienced a more rapid darkening of the tongue in these areas during the early phase of ICI therapy. Moreover, these tongue lightness changes demonstrated significant associations with both PFS and OS in multivariate analysis, highlighting their dual role as both predictive and prognostic biomarkers. These results support the need for periodic observation of tongue lightness changes in patients with NSCLC receiving ICI therapy.

Certain tongue characteristics, such as a mirror-like appearance, thick coating, and tongue moisture, have been reported in patients with cancer and suggested as potential indicators for screening and early diagnosis via TD (16). However, the findings of the present study cannot be directly compared with those of previous studies, as healthy individuals were not included as controls, and the digital TD system used did not assess features such as mirror-like tongue or moisture. A previous cross-sectional study also suggested that a blue-purple tongue, greasy coating, and thick coating were more frequently observed in patients with stage III and IV lung cancer (14,47). In contrast, most patients with NSCLC in the present study exhibited a normal, light-pink tongue with a thin coating at baseline, suggesting that TD findings may appear nonspecific when assessed at a single time point. This highlights the importance of longitudinal observation in uncovering changes in tongue characteristics.

One limitation of this study is that few prior studies have utilized the same digital TD system for cancer, making it difficult to directly compare our findings with existing literature. Additionally, baseline differences in ICI types and PD-L1 expression levels between the responder and nonresponder groups may have confounded our findings, as the associations with PFS and OS lost statistical significance after additional adjustment, despite showing similar trends. Another limitation is that Visits 4, 5, and 6 of the nonresponder group were excluded from group comparisons owing to an increasing number of follow-up discontinuations over time. To address this issue, we applied a linear mixed-effects model, which allows for the inclusion of incomplete data, to analyze longitudinal changes in TD variables. Our study was conducted exclusively with Korean participants using a Korean-developed TD system, which may limit the generalizability of our findings to diverse populations due to potential variations in tongue morphology and color characteristics across different ethnicities. Despite these limitations, this study is noteworthy for being a longitudinal investigation to track tongue lightness changes during ICI therapy in patients with NSCLC, extending observations to 45 weeks with objective quantification methods. Given the current insufficiency of reliable prognostic biomarkers for ICI therapy, our study provides preliminary evidence that noninvasive tongue lightness monitoring may serve as a complementary prognostic tool, particularly valuable in resource-limited settings where expensive molecular testing may not be readily available.

In real-world practice, diagnoses of traditional medicine patterns and biomedical diseases are often integrated (48). The inclusion of traditional medicine pattern codes in the ICD-11 is expected to promote wider use of traditional diagnostic frameworks. However, accurate PI requires a comprehensive approach that includes patient history, objective signs, and validated questionnaires. TD, as a visible and easily accessible sign, holds value in both traditional and conventional medicine. Changes in tongue characteristics among patients with NSCLC receiving ICIs—especially the early and significant alterations in tongue lightness—should be considered in clinical care. Regular follow-up every 9 weeks using a digital TD system may help objectively monitor changes of the tongue, such as darkening and reddening. When quantitative evaluation is not feasible, using a camera or image-based system is preferable to relying solely on visual inspection. While our study demonstrates associations between tongue lightness changes and survival outcomes, the absence of clearly defined clinical thresholds limits immediate clinical application. Future studies with larger sample sizes and extended follow-up that employ advanced longitudinal modeling methods to address dropout and temporal variability are needed. These studies could evaluate whether tongue parameters provide incremental predictive value beyond established biomarkers through AUC comparison, calibration assessment, and net reclassification improvement analysis. They are needed to establish clinically applicable cutoff values and optimal sensitivity and specificity for patient stratification. Such validation would be crucial for translating these preliminary findings into evidence-based clinical tools that can improve patient outcomes and optimize treatment strategies in NSCLC immunotherapy. Additionally, future studies incorporating healthy control groups would provide valuable baseline comparisons and help establish normative tongue characteristics, which would enhance the interpretation of tongue changes in cancer patients and strengthen the clinical utility of digital tongue diagnosis as a monitoring tool.

## 5 Conclusion

This prospective, multicenter, observational study collected digital TD data from patients with NSCLC receiving ICIs and demonstrated that treatment response was associated with distinct temporal changes in tongue lightness parameters over a 45-week period. Notably, nonresponders showed significantly greater decreases in tongue lightness (darker tongues) over time in body, fur, root, and center regions during early treatment phases. Multivariate Cox proportional hazards analysis revealed that increases in tongue lightness, particularly in the body and center regions, were significantly associated with PFS and increases in fur area lightness were associated with longer OS. These findings provide preliminary evidence for incorporating digital TD into routine clinical monitoring of patients with NSCLC receiving immunotherapy, potentially offering a cost-effective complement to existing biomarkers.

## Data Availability

The datasets analyzed during the current study are available from the corresponding authors upon reasonable request.
