# Supplementary material for: Temporal changes in tongue color during immune checkpoint inhibitor therapy in patients with non-small-cell lung cancer: a prospective observational study using digital tongue diagnosis
Source: Oncol Rev. 2025 Dec 9;19:1697252. doi: 10.3389/or.2025.1697252 (PMC12722973; doi:10.3389/or.2025.1697252)
Supplement: Supplementary file 1 [file Supplementaryfile1.docx]

Additional file 1. Mean values of digital tongue diagnosis variables across visits

| Variables | Visit 1 | Visit 2 | Visit 3 | Visit 4 | Visit 5 | Visit 6 |
| --- | --- | --- | --- | --- | --- | --- |
| Body L | 53.6±0.278 (53.0–54.1) | 52.4±0.312 (51.8–53.0) | 53.2±0.388 (52.5–54.0) | 52.7±0.443 (51.8–53.5) | 53.6±0.514 (52.6–54.6) | 53.1±0.574 (52.0–54.3) |
| Body a | 25.3±0.291 (24.8–25.9) | 27.0±0.326 (26.4–27.7) | 27.2±0.405 (26.4–27.9) | 26.9±0.461 (26.0–27.8) | 27.1±0.535 (26.0–28.1) | 28.1±0.597 (26.9–29.2) |
| Body b | 11.1±0.200 (10.8–11.5) | 10.9±0.225 (10.4–11.3) | 10.8±0.282 (10.3–11.4) | 10.6±0.322 (10.0–11.2) | 10.7±0.375 (10.0–11.4) | 11.6±0.419 (10.7–12.4) |
| Fur L | 51.1±0.589 (49.9–52.3) | 48.1±0.678 (46.8–49.5) | 48.6±0.884 (46.9–50.4) | 50.0±1.030 (48.0–52.0) | 50.1±1.220 (47.7–52.5) | 50.3±1.380 (47.6–53.0) |
| Fur a | 13.0±0.233 (12.5–13.4) | 13.9±0.267 (13.4–14.4) | 13.7±0.347 (13.0–14.4) | 13.7±0.403 (12.9–14.5) | 13.8±0.476 (12.9–14.8) | 14.4±0.536 (13.4–15.5) |
| Fur b | 11.6±0.269 (11.1–12.4) | 10.9±0.310 (10.3–11.5) | 10.7±0.403 (9.9–11.4) | 10.9±0.469 (10.0–11.8) | 11.0±0.555 (9.9–12.1) | 11.6±0.626 (10.4–12.9) |
| Root L | 51.4±0.430 (50.5–52.2) | 50.1±0.484 (49.1–51.1) | 50.7±0.606 (49.5–51.9) | 50.1±0.693 (48.7–51.5) | 51.0±0.807 (49.4–52.6) | 50.5±0.902 (48.7–52.3) |
| Root a | 18.6±0.308 (18.0–19.2) | 20.2±0.346 (19.5–20.9) | 20.3±0.433 (19.5–21.2) | 19.5±0.495 (18.6–20.5) | 19.9±0.576 (18.7–21.0) | 20.1±0.644 (18.8–21.4) |
| Root b | 11.1±0.244 (10.6–11.6) | 10.6±0.283 (10.0–11.2) | 10.3±0.372 (9.6–11.0) | 10.1±0.435 (9.3–11.0) | 10.3±0.516 (9.3–11.3) | 10.9±0.584 (9.7–12.0) |
| Center L | 56.6±0.340 (55.9–57.3) | 55.0±0.382 (54.3–55.8) | 55.9±0.476 (55.0–56.9) | 55.6±0.543 (54.5–56.6) | 56.5±0.631 (55.3–57.7) | 55.9±0.705 (54.6–57.3) |
| Center a | 23.0±0.351 (22.3–23.7) | 25.0±0.396 (24.2–25.7) | 24.9±0.499 (23.9–25.9) | 24.3±0.572 (23.2–25.4) | 24.7±0.667 (23.3–26.0) | 25.8±0.747 (24.3–27.3) |
| Center b | 11.3±0.233 (10.8–11.7) | 10.7±0.264 (10.2–11.2) | 10.7±0.335 (10.0–11.3) | 10.5±0.386 (9.8–11.3) | 10.6±0.452 (9.7–11.5) | 11.6±0.507 (10.6–12.6) |
| Side L | 52.0±0.309 (51.4–52.6) | 51.1±0.348 (50.4–51.8) | 51.9±0.438 (51.0–52.8) | 51.4±0.503 (50.4–52.3) | 52.4±0.587 (51.2–53.5) | 51.9±0.656 (50.6–53.2) |
| Side a | 26.9±0.357 (26.2–27.6) | 28.8±0.396 (28.0–29.6) | 29.0±0.485 (28.0–29.9) | 28.6±0.548 (27.6–29.7) | 28.7±0.633 (27.5–30.0) | 29.9±0.703 (28.5–31.3) |
| Side b | 11.9±0.207 (11.5–12.3) | 12.0±0.227 (11.5–12.4) | 12.1±0.272 (11.6–12.7) | 11.9±0.305 (11.3–12.5) | 12.2±0.349 (11.5–12.9) | 12.7±0.386 (12.0–13.5) |
| Tip L | 50.9±0.351 (50.2–51.6) | 50.0±0.395 (49.2–50.7) | 50.9±0.493 (49.9–51.8) | 50.2±0.563 (49.1–51.3) | 50.8±0.655 (49.5–52.1) | 50.4±0.731 (48.9–51.8) |
| Tip a | 28.6±0.399 (27.8–29.4) | 30.7±0.447 (29.9–31.6) | 31.0±0.554 (29.9–32.1) | 30.2±0.631 (29.0–31.5) | 30.3±0.733 (28.9–31.8) | 31.8±0.817 (30.2–33.4) |
| Tip b | 11.8±0.209 (11.4–12.2) | 11.8±0.234 (11.4–12.3) | 12.1±0.289 (11.5–12.7) | 11.5±0.329 (10.9–12.2) | 12.0±0.381 (11.2–12.7) | 12.4±0.425 (11.6–13.3) |
| Toothmark | 9.9±0.506 (8.9–10.9) | 10.0±0.597 (8.8–11.2) | 11.0±0.816 (9.4–12.6) | 10.6±0.974 (8.7–12.5) | 10.7±1.180 (8.4–13.0) | 10.5±1.350 (7.8–13.1) |

Data are presented as mean±standard error (95% confidence interval).
