# Supplementary material for: Temporal changes in tongue color during immune checkpoint inhibitor therapy in patients with non-small-cell lung cancer: a prospective observational study using digital tongue diagnosis
Source: Oncol Rev. 2025 Dec 9;19:1697252. doi: 10.3389/or.2025.1697252 (PMC12722973; doi:10.3389/or.2025.1697252)
Supplement: Supplementary file 2 [file Supplementaryfile3.docx]

Additional file 3. Changes in tongue diagnosis variables between response and non-response groups during visits 1 to 3

Table S3-1. Comparison of digital tongue diagnosis variable changes between responders and non-responders during early ICI treatment (Visit 1–3)

| Variable | Fixed effects | Estimate | Standard error | t-value | *p*-value | Random effects | | |
| --- | --- | --- | --- | --- | --- | --- | --- | --- |
|  |  |  |  |  |  | Patient variance | Residual variance | ICC |
| Body L | (Intercept) | 51.39 | 1.92 | 26.76 | <0.001* | 12.07 | 4.27 | 0.74 |
|  | Visit | -0.91 | 0.26 | -3.56 | <0.001* |  |  |  |
|  | Response | -1.65 | 0.90 | -1.83 | 0.070 |  |  |  |
|  | Visit×Response | 1.01 | 0.37 | 2.76 | 0.007* |  |  |  |
| Body a | (Intercept) | 24.58 | 2.03 | 12.11 | <0.001* | 8.17 | 4.27 | 0.66 |
|  | Visit | 1.31 | 0.25 | 5.12 | <0.001* |  |  |  |
|  | Response | -0.07 | 0.83 | -0.09 | 0.929 |  |  |  |
|  | Visit×Response | -0.40 | 0.35 | -1.13 | 0.264 |  |  |  |
| Body b | (Intercept) | 6.17 | 1.25 | 4.94 | <0.001* | 6.21 | 2.07 | 0.75 |
|  | Visit | -0.05 | 0.19 | -0.26 | 0.800 |  |  |  |
|  | Response | 1.11 | 0.64 | 1.73 | 0.085 |  |  |  |
|  | Visit×Response | -0.67 | 0.28 | -1.24 | 0.220 |  |  |  |
| Fur L | (Intercept) | 48.41 | 4.03 | 12.02 | <0.001* | 11.86 | 27.78 | 0.30 |
|  | Visit | -3.21 | 0.66 | -4.82 | <0.001* |  |  |  |
|  | Response | -3.57 | 1.76 | -2.03 | 0.044* |  |  |  |
|  | Visit×Response | 2.90 | 0.92 | 3.15 | 0.002* |  |  |  |
| Fur a | (Intercept) | 14.26 | 1.51 | 9.47 | <0.001* | 1.58 | 3.90 | 0.29 |
|  | Visit | 0.43 | 0.25 | 1.71 | 0.090 |  |  |  |
|  | Response | -0.33 | 0.66 | -0.50 | 0.617 |  |  |  |
|  | Visit×Response | 0.17 | 0.35 | 0.49 | 0.623 |  |  |  |
| Fur b | (Intercept) | 6.75 | 1.65 | 4.10 | <0.001* | 10.87 | 5.26 | 0.67 |
|  | Visit | -0.73 | 0.29 | -2.54 | 0.012* |  |  |  |
|  | Response | 0.57 | 0.93 | 0.62 | 0.536 |  |  |  |
|  | Visit×Response | 0.26 | 0.42 | 0.61 | 0.545 |  |  |  |
| Root L | (Intercept) | 46.64 | 2.97 | 15.69 | <0.001* | 20.11 | 9.91 | 0.67 |
|  | Visit | -1.44 | 0.42 | -3.45 | <0.001* |  |  |  |
|  | Response | -3.29 | 1.28 | -2.56 | 0.012* |  |  |  |
|  | Visit×Response | 1.63 | 0.59 | 2.78 | 0.007* |  |  |  |
| Root a | (Intercept) | 19.96 | 2.09 | 9.55 | <0.001* | 6.57 | 5.28 | 0.55 |
|  | Visit | 1.26 | 0.28 | 4.45 | <0.001* |  |  |  |
|  | Response | 0.23 | 0.86 | 0.27 | 0.784 |  |  |  |
|  | Visit×Response | -0.49 | 0.39 | -1.26 | 0.211 |  |  |  |
| Root b | (Intercept) | 4.38 | 1.43 | 3.07 | 0.003* | 10.72 | 4.32 | 0.71 |
|  | Visit | -0.48 | 0.28 | -1.73 | 0.086 |  |  |  |
|  | Response | 0.85 | 0.88 | 0.97 | 0.333 |  |  |  |
|  | Visit×Response | -0.08 | 0.42 | -0.20 | 0.842 |  |  |  |
| Center L | (Intercept) | 53.70 | 2.33 | 23.06 | <0.001* | 17.49 | 6.57 | 0.73 |
|  | Visit | -1.33 | 0.31 | -4.29 | <0.001* |  |  |  |
|  | Response | -2.01 | 1.10 | -1.83 | 0.069 |  |  |  |
|  | Visit×Response | 1.38 | 0.44 | 3.12 | 0.003* |  |  |  |
| Center a | (Intercept) | 24.98 | 2.43 | 10.28 | <0.001* | 14.03 | 6.44 | 0.69 |
|  | Visit | 1.58 | 0.33 | 4.84 | <0.001* |  |  |  |
|  | Response | 0.36 | 1.05 | 0.35 | 0.730 |  |  |  |
|  | Visit×Response | -0.67 | 0.46 | -1.46 | 0.148 |  |  |  |
| Center b | (Intercept) | 5.82 | 1.36 | 4.28 | <0.001* | 10.19 | 2.94 | 0.78 |
|  | Visit | -0.27 | 0.22 | -1.21 | 0.228 |  |  |  |
|  | Response | 1.38 | 0.79 | 1.76 | 0.080 |  |  |  |
|  | Visit×Response | -0.29 | 0.33 | -0.87 | 0.389 |  |  |  |
| Side L | (Intercept) | 49.83 | 2.05 | 24.35 | <0.001* | 17.65 | 5.72 | 0.76 |
|  | Visit | -0.75 | 0.30 | -2.45 | 0.016* |  |  |  |
|  | Response | -1.49 | 1.06 | -1.40 | 0.164 |  |  |  |
|  | Visit×Response | 0.85 | 0.44 | 1.92 | 0.058 |  |  |  |
| Side a | (Intercept) | 23.87 | 2.44 | 9.79 | <0.001* | 8.32 | 5.89 | 0.59 |
|  | Visit | 1.48 | 0.31 | 4.82 | <0.001* |  |  |  |
|  | Response | -0.27 | 0.93 | -0.29 | 0.771 |  |  |  |
|  | Visit×Response | -0.41 | 0.42 | -0.97 | 0.333 |  |  |  |
| Side b | (Intercept) | 7.29 | 1.41 | 5.18 | <0.001* | 5.15 | 1.70 | 0.75 |
|  | Visit | 0.20 | 0.17 | 1.12 | 0.265 |  |  |  |
|  | Response | 0.31 | 0.58 | 0.53 | 0.596 |  |  |  |
|  | Visit×Response | -0.17 | 0.25 | -0.69 | 0.490 |  |  |  |
| Tip L | (Intercept) | 47.28 | 2.40 | 19.73 | <0.001* | 22.90 | 6.45 | 0.78 |
|  | Visit | -0.68 | 0.33 | -2.03 | 0.045* |  |  |  |
|  | Response | -0.77 | 1.18 | -0.65 | 0.516 |  |  |  |
|  | Visit×Response | 0.67 | 0.48 | 1.38 | 0.171 |  |  |  |
| Tib a | (Intercept) | 30.11 | 2.77 | 10.88 | <0.001* | 24.16 | 7.14 | 0.77 |
|  | Visit | 1.70 | 0.36 | 4.69 | <0.001* |  |  |  |
|  | Response | -0.42 | 1.23 | -0.34 | 0.733 |  |  |  |
|  | Visit×Response | -0.45 | 0.52 | -0.88 | 0.384 |  |  |  |
| Tip b | (Intercept) | 9.30 | 1.42 | 6.57 | <0.001* | 5.82 | 2.27 | 0.72 |
|  | Visit | 0.15 | 0.20 | 0.72 | 0.471 |  |  |  |
|  | Response | 0.38 | 0.65 | 0.59 | 0.556 |  |  |  |
|  | Visit×Response | -0.10 | 0.29 | -0.35 | 0.727 |  |  |  |

Interaction terms (Visit × Response) were included to test whether the pattern of change over time differed between responders and non-responders. Intercept: mean outcome at Visit 1 in the responder group; Visit: time effect within the responder group; Response: group difference at Visit 1; Visit × Response: interaction term indicating group differences in change over time; Patient variance: inter-individual variability; Residual variance: intra-individual variability; ICC: intraclass correlation coefficient.

Figure S3-1. Changes in tongue diagnosis variables between response and non-response groups during visits 1 to 3

| 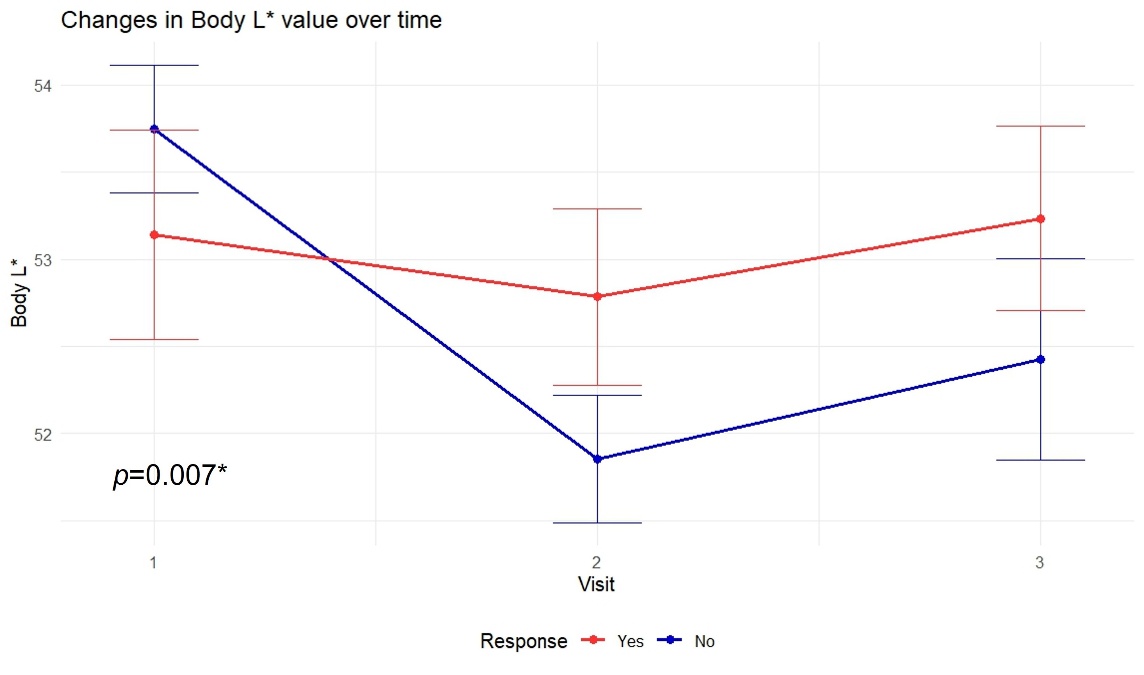 | 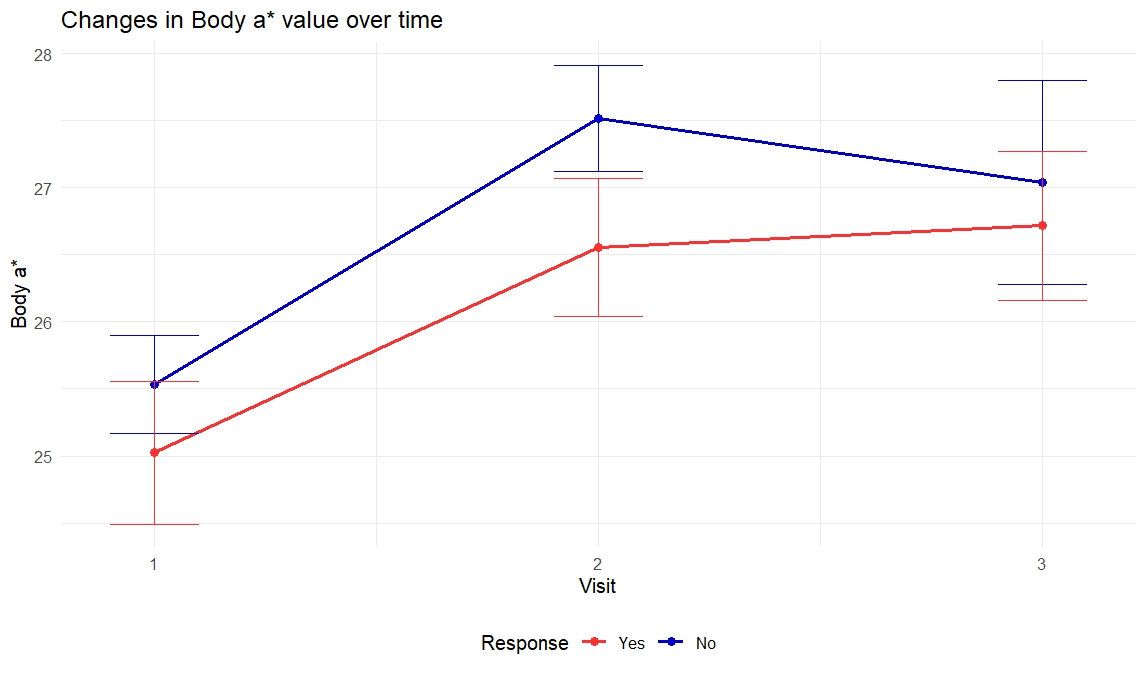 |
| --- | --- |
| 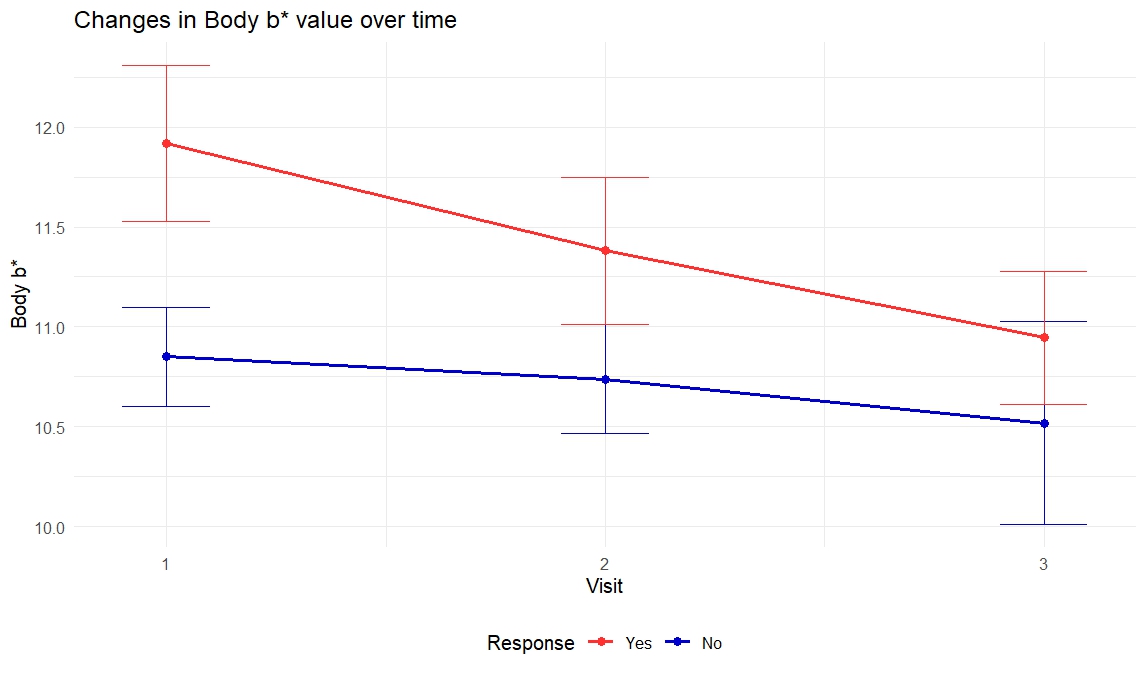 | 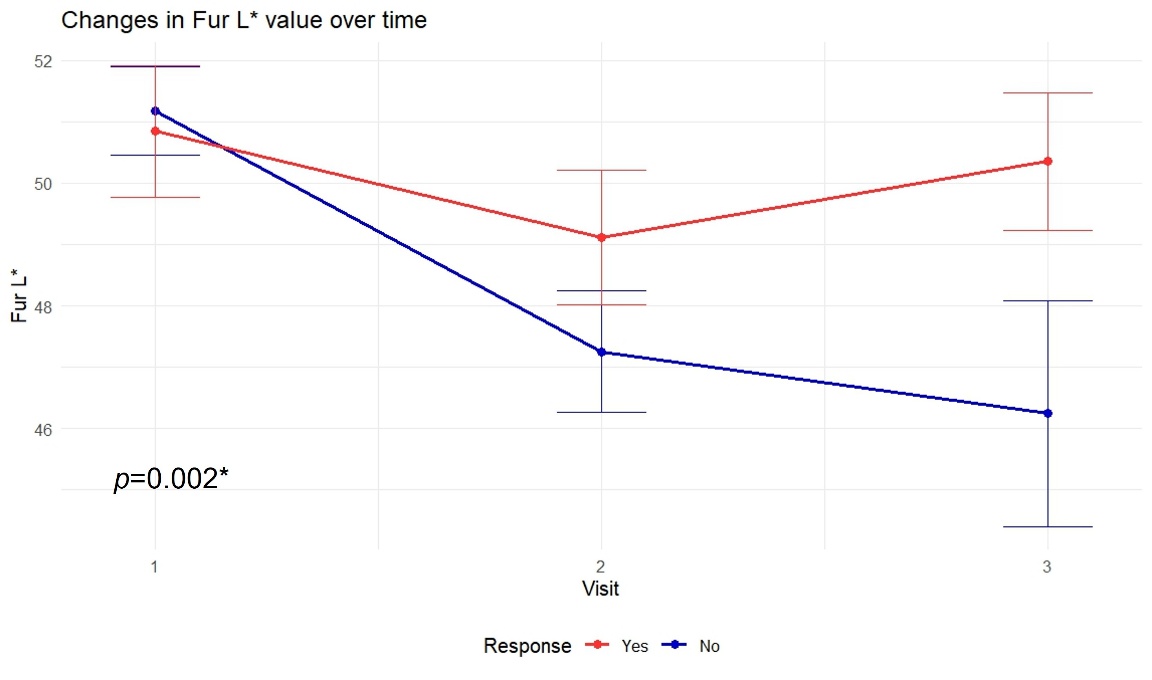 |
| 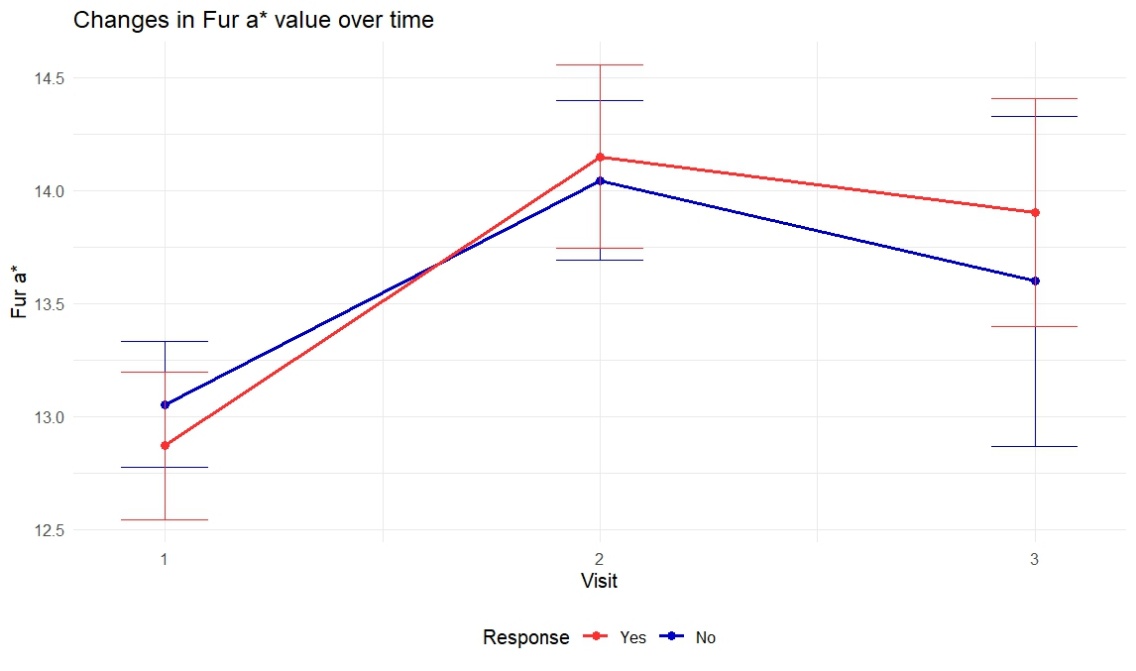 | 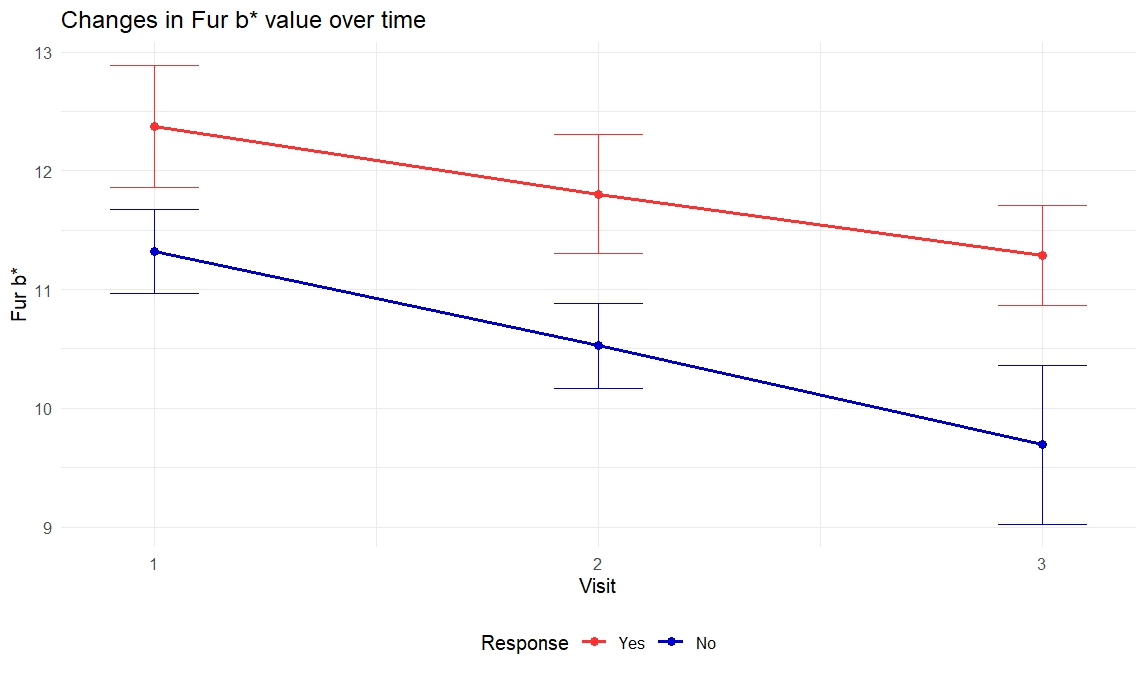 |
| 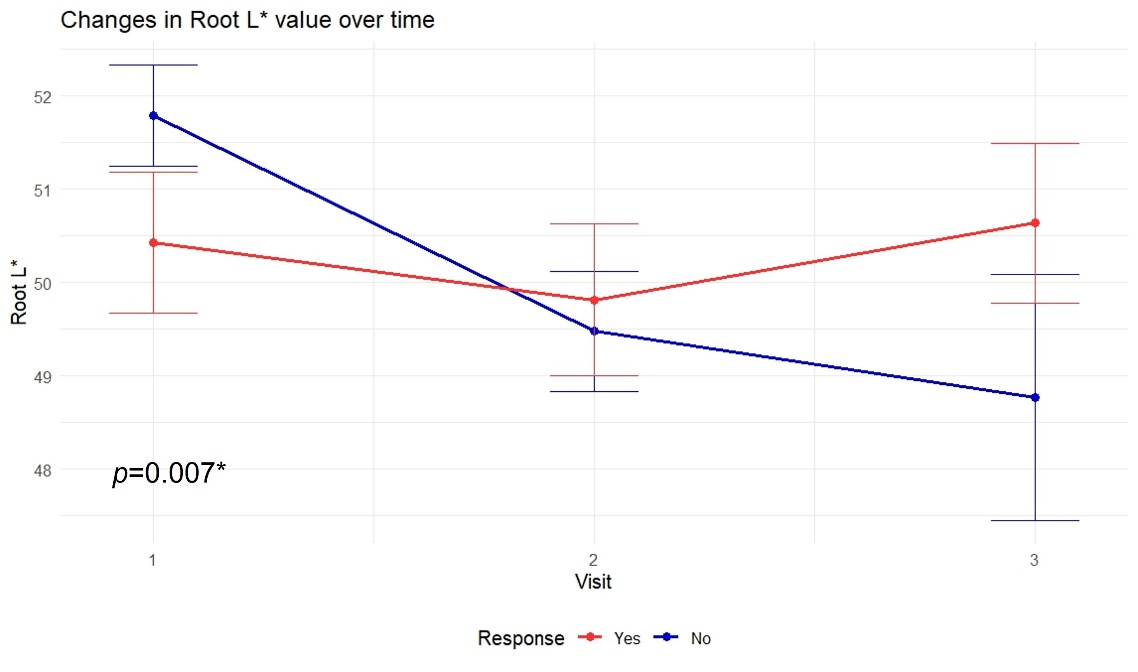 | 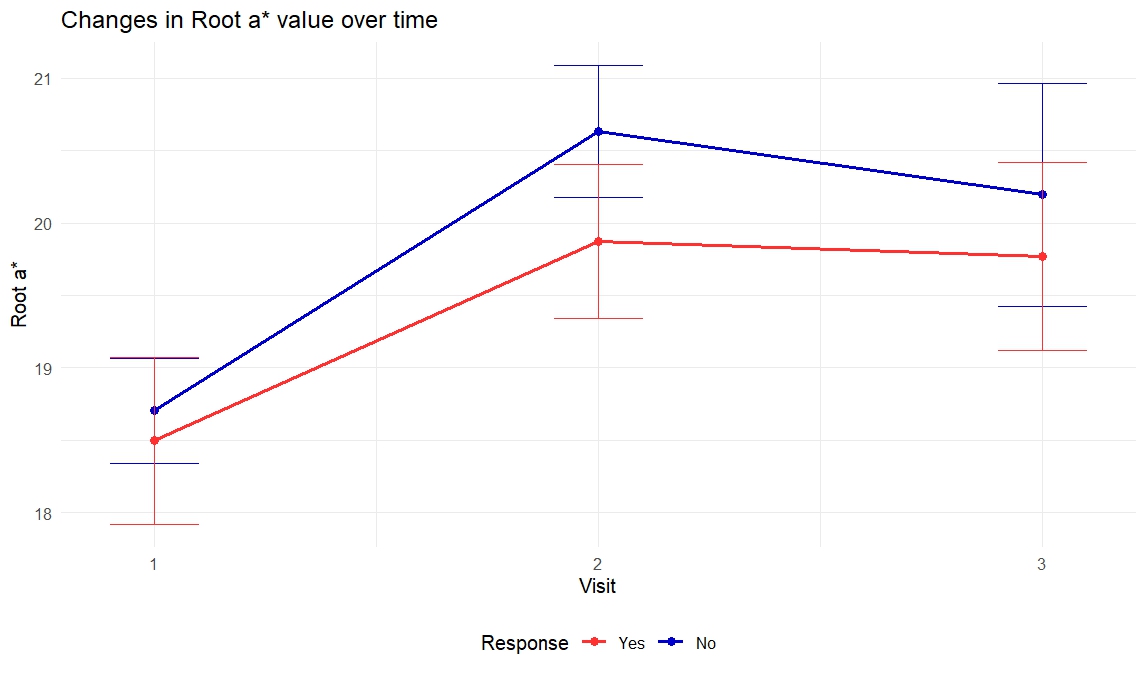 |
| 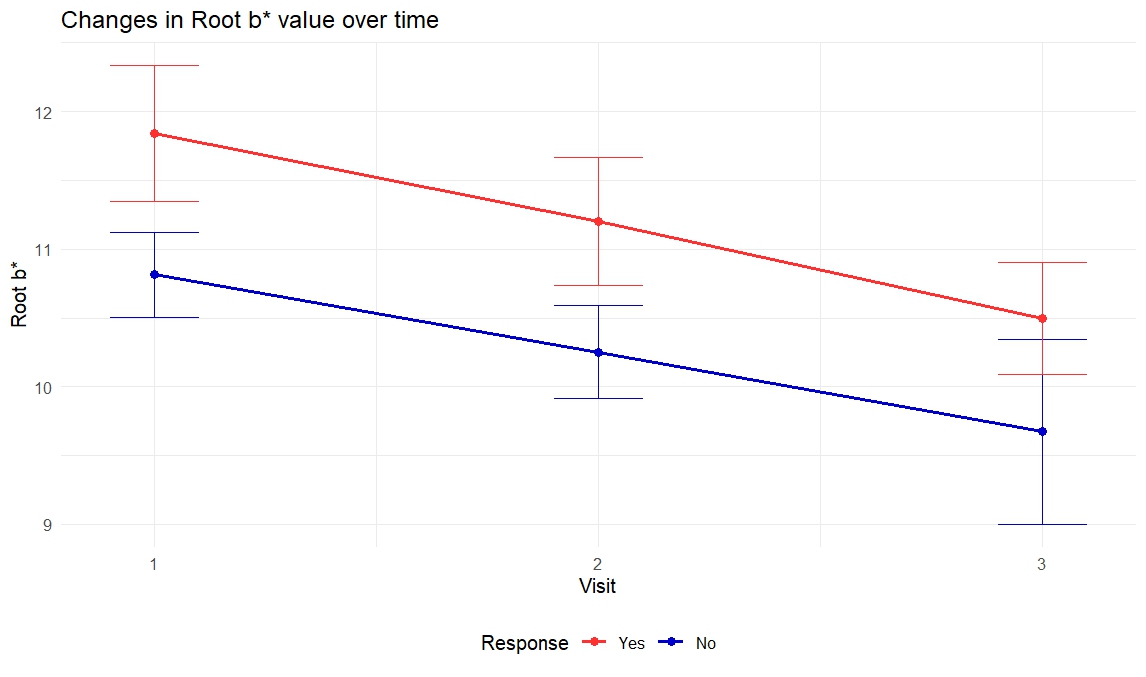 | 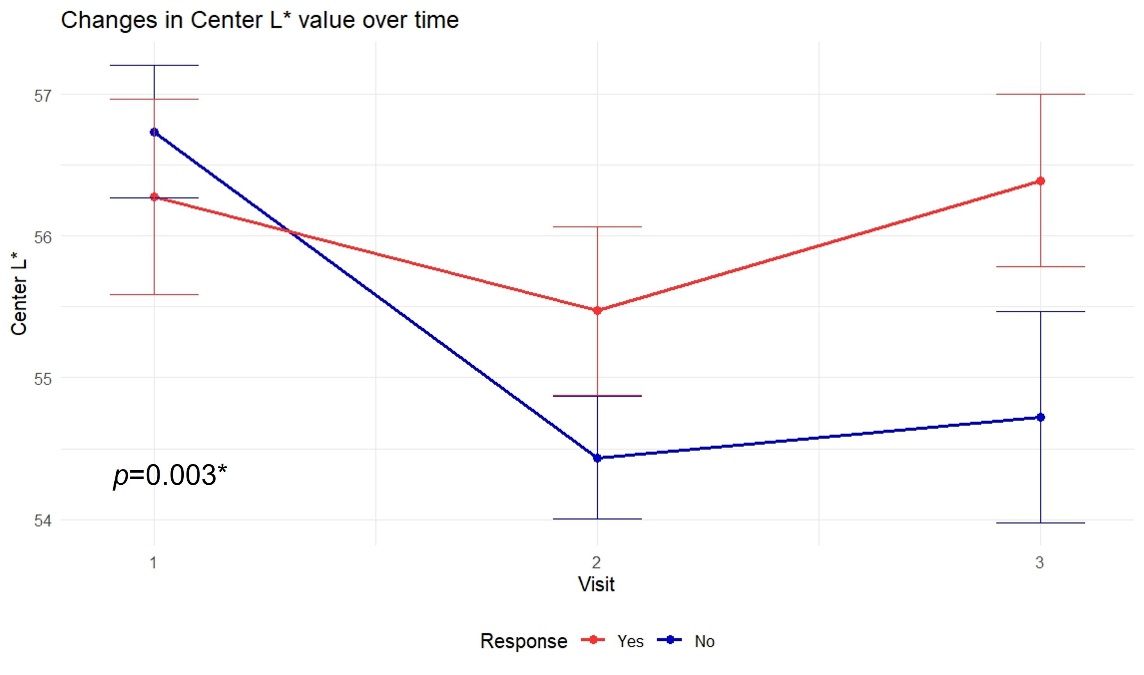 |
| 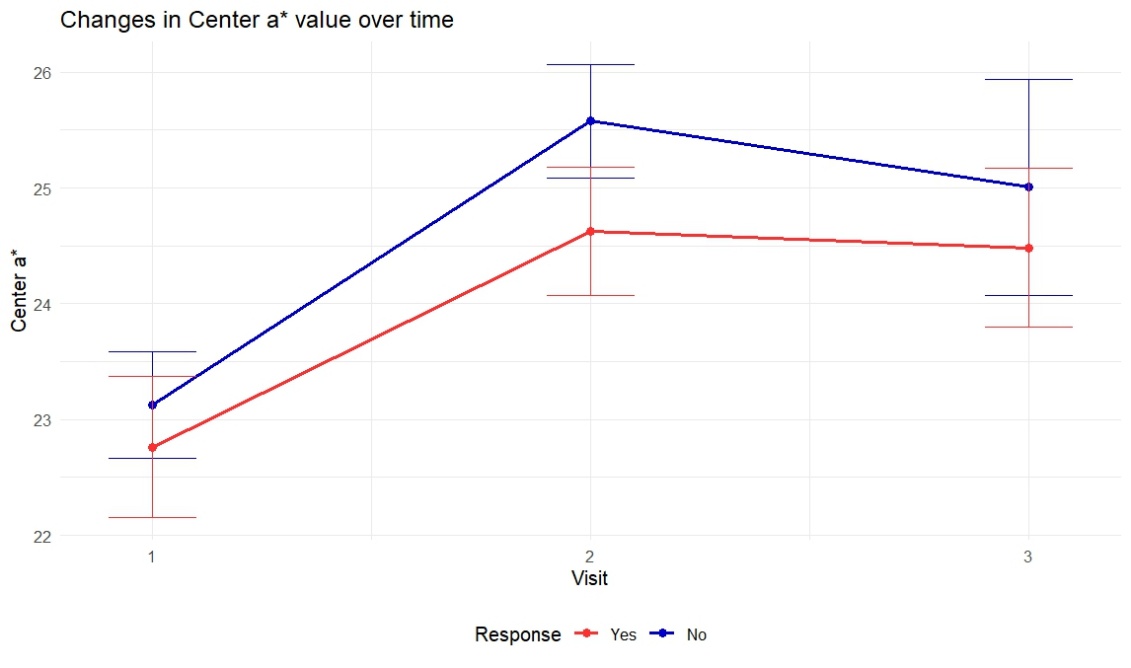 | 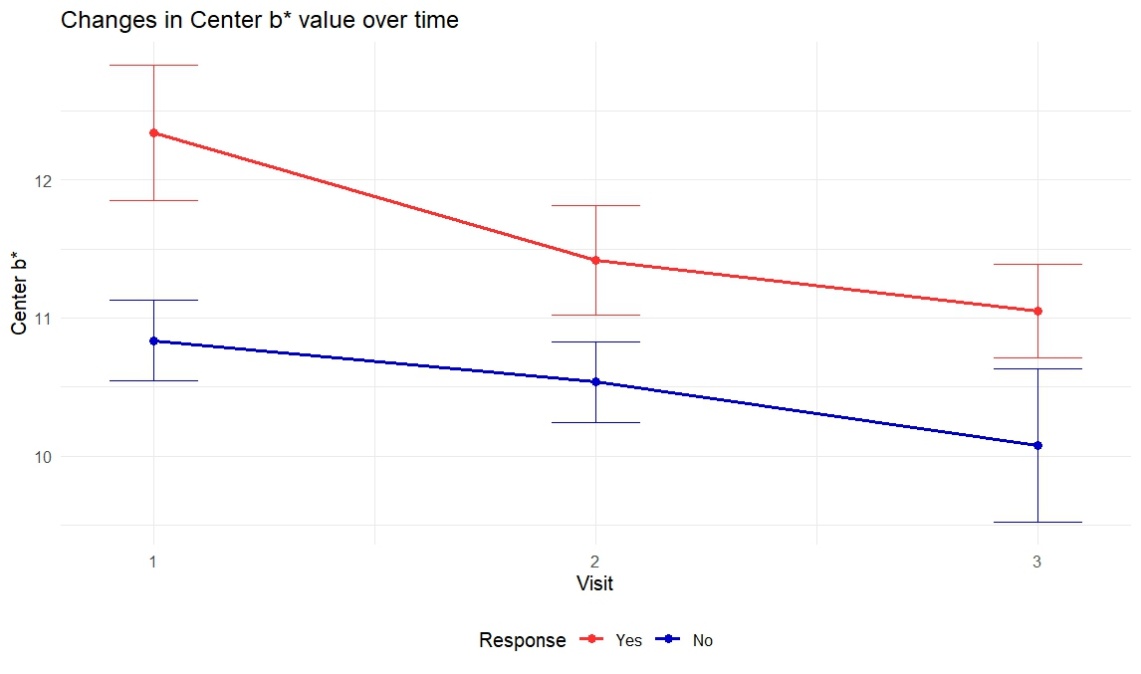 |
| 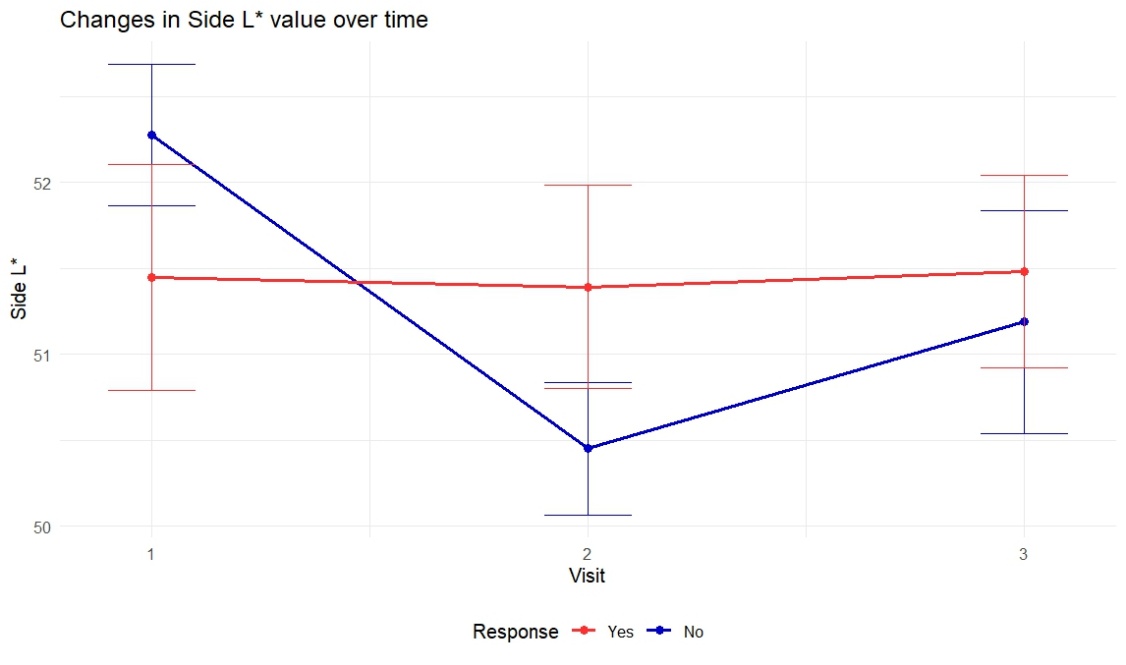 | 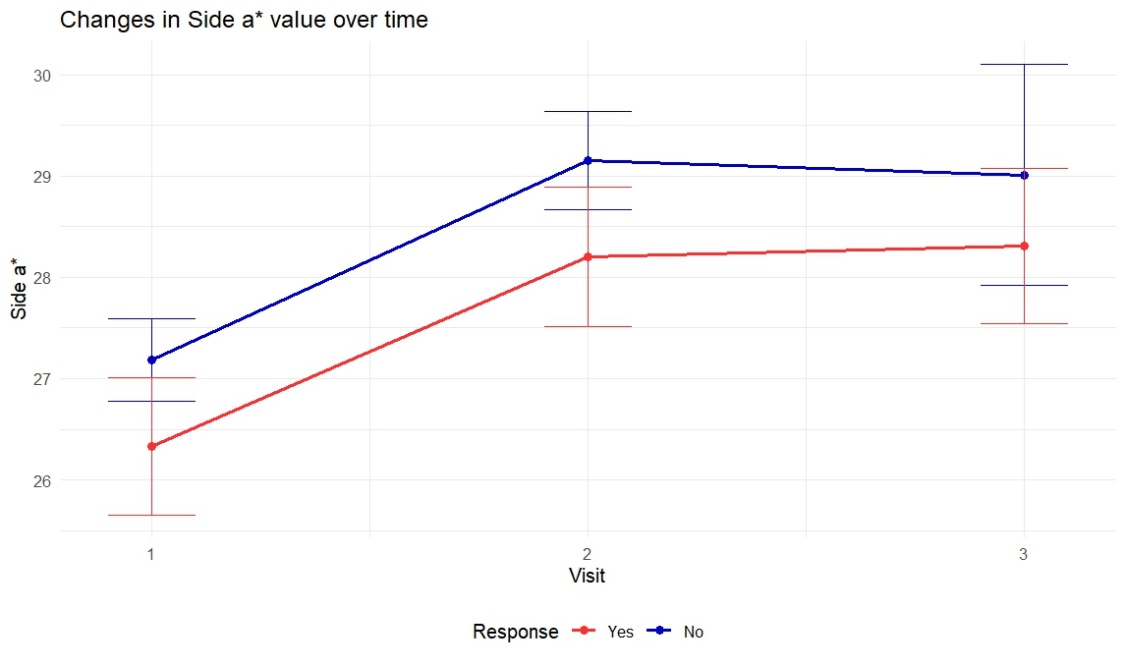 |
| 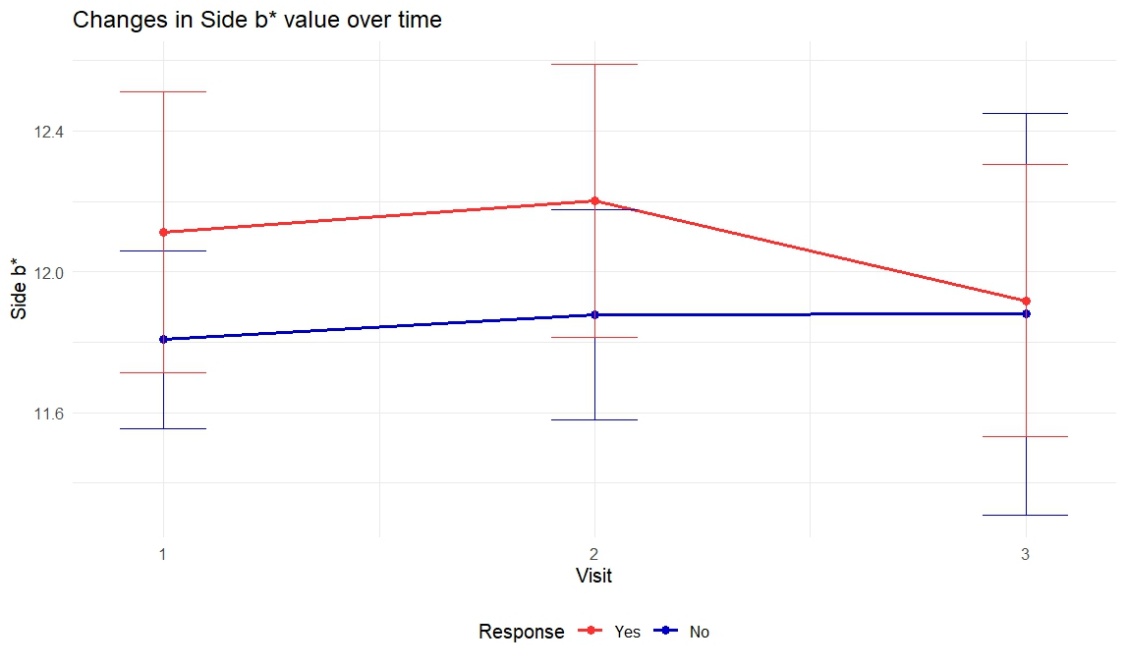 | 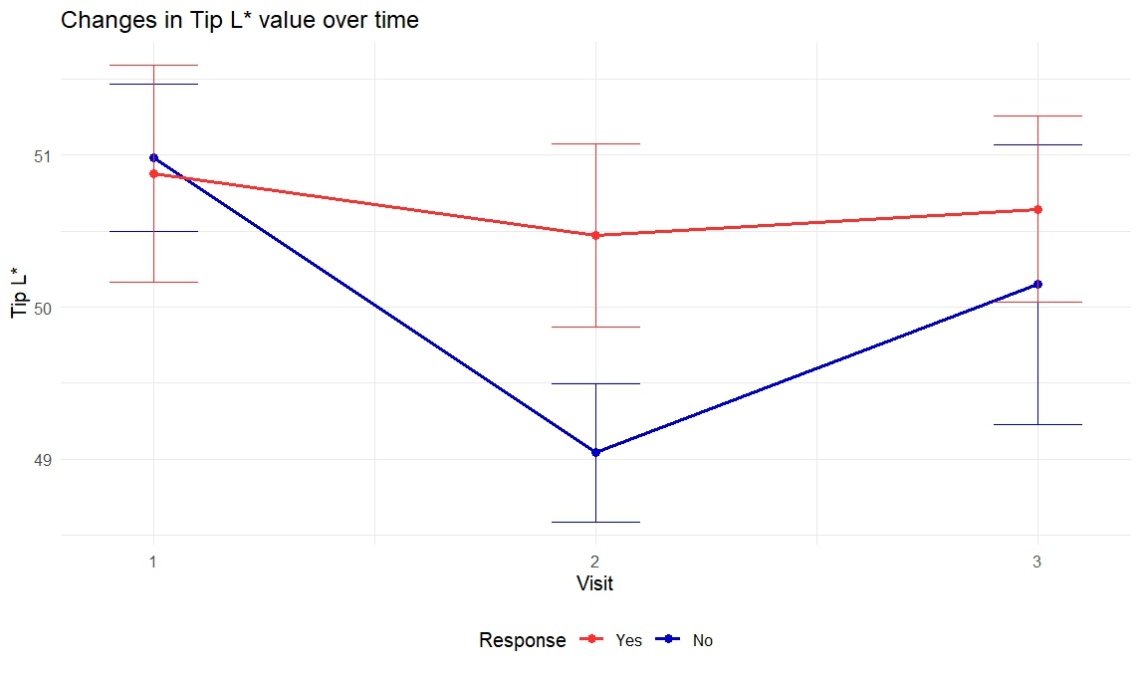 |
| 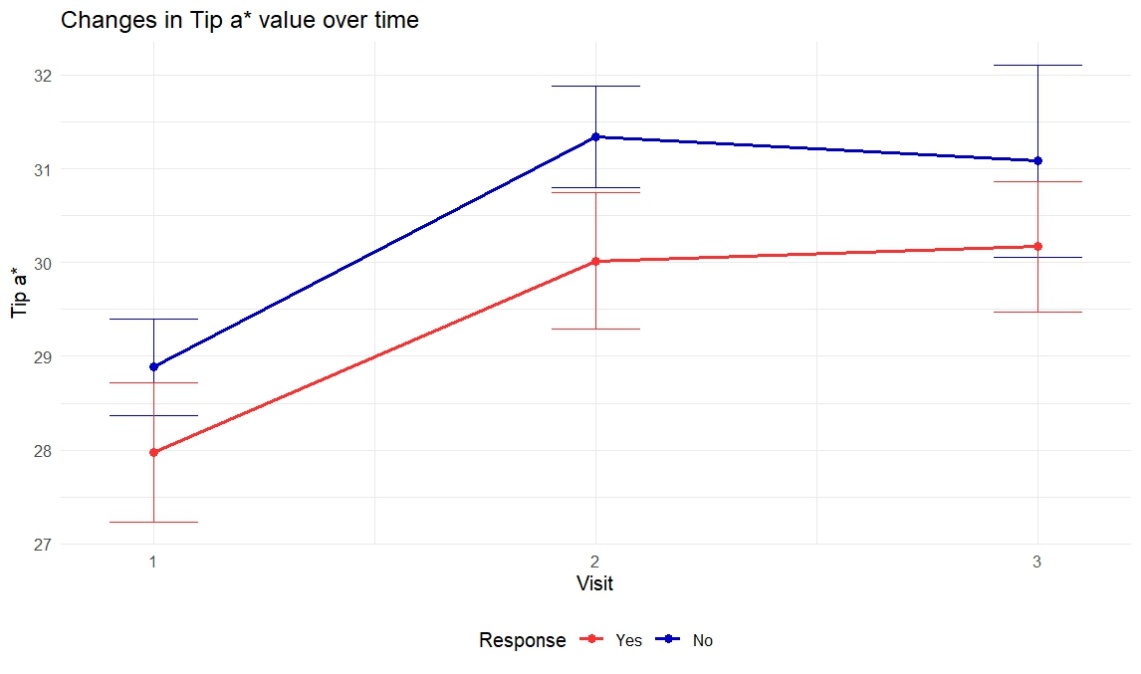 | 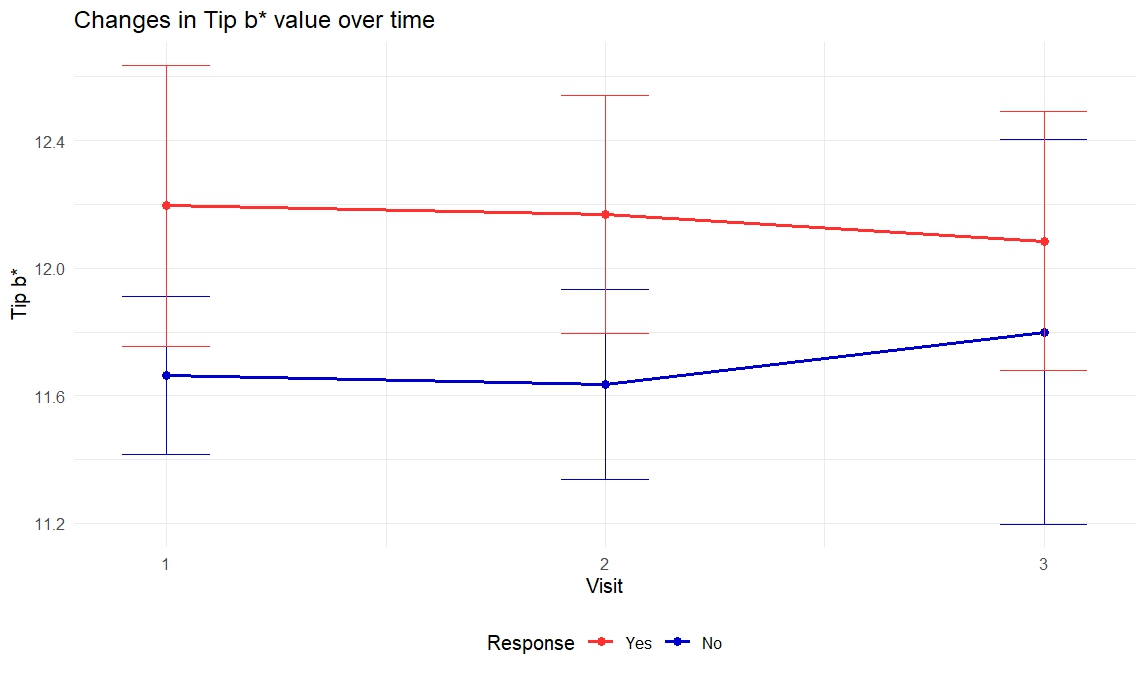 |
