# Supplementary material for: Temporal changes in tongue color during immune checkpoint inhibitor therapy in patients with non-small-cell lung cancer: a prospective observational study using digital tongue diagnosis
Source: Oncol Rev. 2025 Dec 9;19:1697252. doi: 10.3389/or.2025.1697252 (PMC12722973; doi:10.3389/or.2025.1697252)
Supplement: Supplementary file 3 [file Supplementaryfile2.docx]

Additional file 2. Comparative analysis of digital tongue diagnosis variables against baseline values during immune checkpoint inhibitor therapy

Table S2-1. Changes of tongue diagnosis variables compared to visit 1

| Variables | Visits 1 and 2 | | Visits 1 and 3 | | Visits 1 and 4 | | Visits 1 and 5 | | Visits 1 and 6 | |
| --- | --- | --- | --- | --- | --- | --- | --- | --- | --- | --- |
|  | MD | *p*-value | MD | *p*-value | MD | *p*-value | MD | *p*-value | MD | *p*-value |
| Body L | -1.13 | 0.001* | -0.33 | 0.945 | -0.89 | 0.299 | 0.01 | >0.999 | -0.43 | 0.974 |
| Body a | 1.70 | <0.001* | 1.81 | <0.001* | 1.52 | 0.009* | 1.72 | 0.013* | 2.72 | <0.001* |
| Body b | -0.26 | 0.811 | -0.31 | 0.864 | -0.55 | 0.502 | -0.43 | 0.846 | 0.43 | 0.907 |
| Fur L | -2.99 | <0.001* | -2.45 | 0.080 | -1.11 | 0.899 | -1.01 | 0.965 | -0.78 | 0.993 |
| Fur a | 0.93 | 0.012* | 0.77 | 0.248 | 0.75 | 0.453 | 0.88 | 0.454 | 1.45 | 0.082 |
| Fur b | -0.67 | 0.301 | -0.95 | 0.197 | -0.71 | 0.680 | -0.58 | 0.909 | 0.01 | >0.999 |
| Root L | -1.27 | 0.060 | -0.71 | 0.830 | -1.27 | 0.406 | -0.40 | 0.996 | -0.85 | 0.929 |
| Root a | 1.60 | <0.001* | 1.72 | <0.001* | 0.94 | 0.365 | 1.28 | 0.212 | 1.50 | 0.167 |
| Root b | -0.49 | 0.585 | -0.79 | 0.324 | -0.95 | 0.287 | -0.81 | 0.640 | -0.23 | 0.999 |
| Center L | -1.60 | <0.001* | -0.68 | 0.666 | -1.05 | 0.337 | -0.12 | >0.999 | -0.67 | 0.928 |
| Center a | 1.99 | <0.001* | 1.93 | 0.001* | 1.31 | 0.179 | 1.68 | 0.111 | 2.81 | 0.002* |
| Center b | -0.53 | 0.315 | -0.58 | 0.500 | -0.74 | 0.380 | -0.62 | 0.732 | 0.34 | 0.985 |
| Side L | -0.94 | 0.055 | -0.12 | >0.999 | -0.67 | 0.743 | 0.36 | 0.990 | -0.10 | >0.999 |
| Side a | 1.88 | <0.001* | 2.07 | <0.001* | 1.74 | 0.011* | 1.84 | 0.030* | 3.02 | <0.001* |
| Side b | 0.09 | 0.996 | 0.27 | 0.867 | -0.02 | >0.999 | 0.29 | 0.950 | 0.87 | 0.161 |
| Tip L | -0.98 | 0.086 | -0.06 | >0.999 | -0.73 | 0.763 | -0.14 | 0.999 | -0.55 | 0.973 |
| Tip a | 2.18 | <0.001* | 2.43 | <0.001* | 1.66 | 0.070 | 1.77 | 0.131 | 3.20 | 0.001* |
| Tip b | 0.04 | >0.9999 | 0.32 | 0.8427 | 0.26 | 0.9594 | 0.18 | 0.9964 | 0.63 | 0.6535 |
| Toothmark | 0.1 | >0.999 | 1.10 | 0.841 | 0.68 | 0.988 | 0.78 | 0.990 | 0.56 | 0.999 |

MD, Mean difference. **p*<0.05.

Figure S2-1. Individual and mean changes in variables during visits 1 to 6.

| 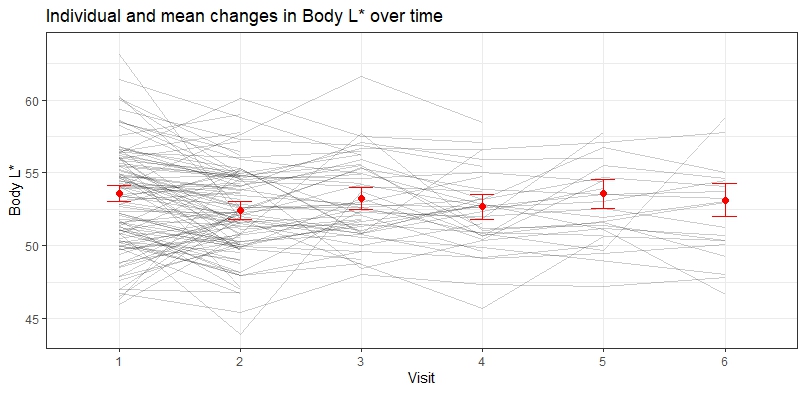 | 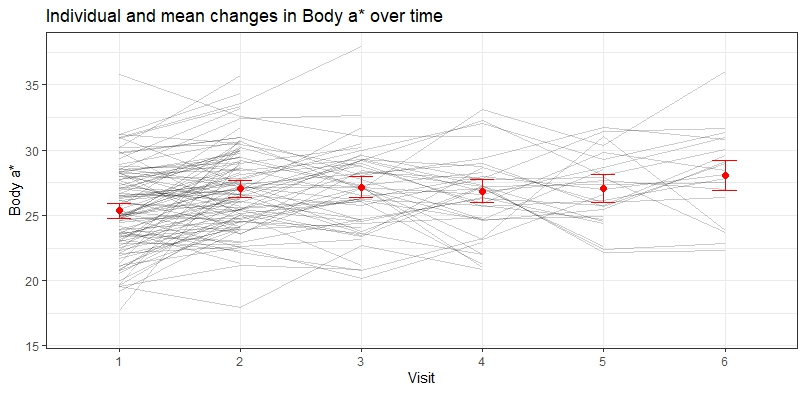 |
| --- | --- |
| 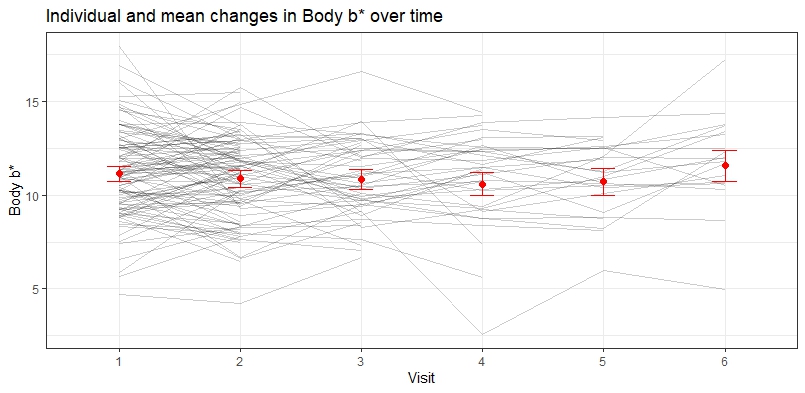 | 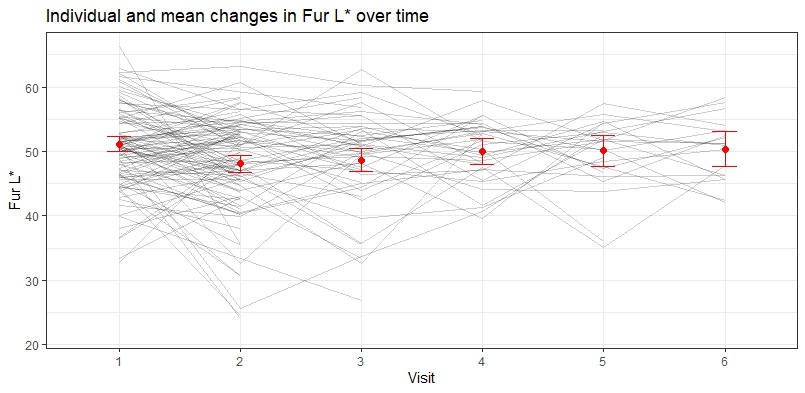 |
| 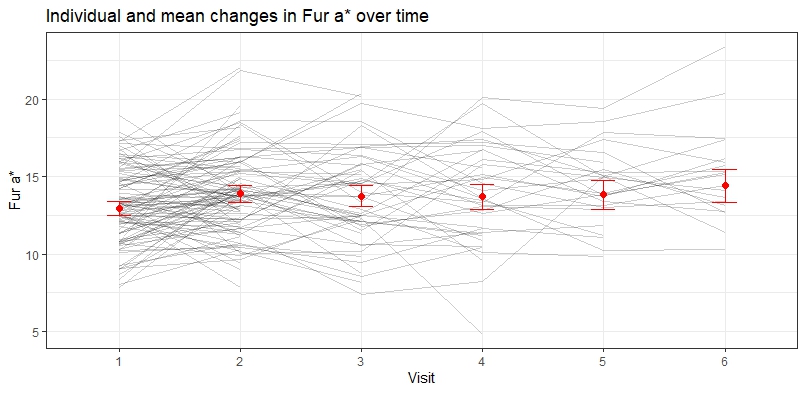 | 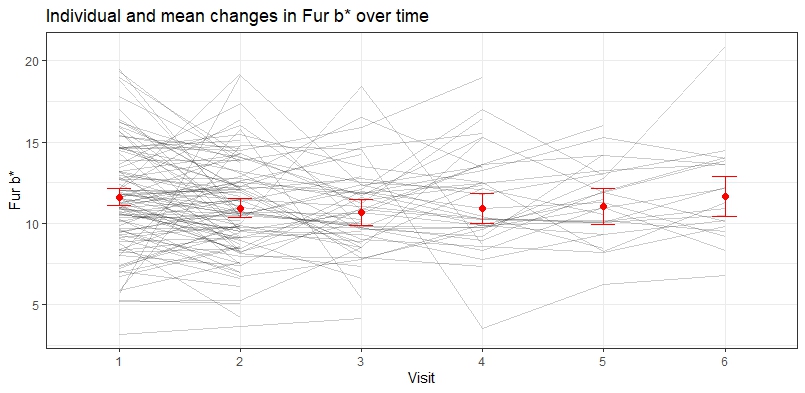 |
| 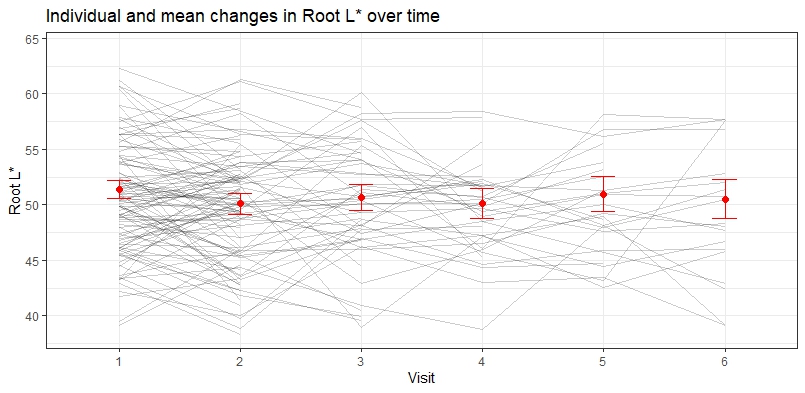 | 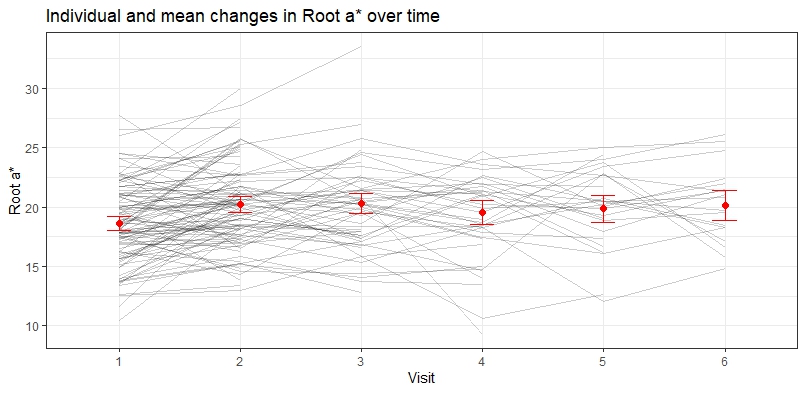 |
| 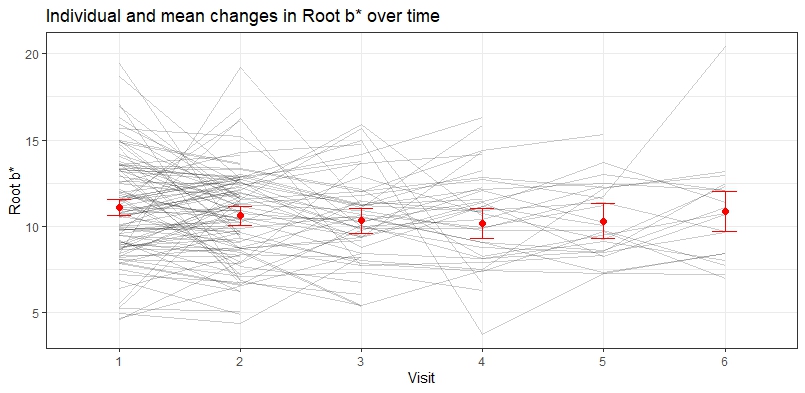 | 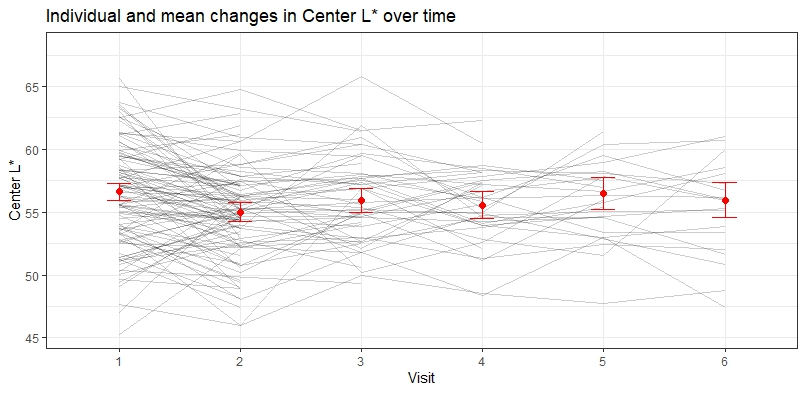 |
| 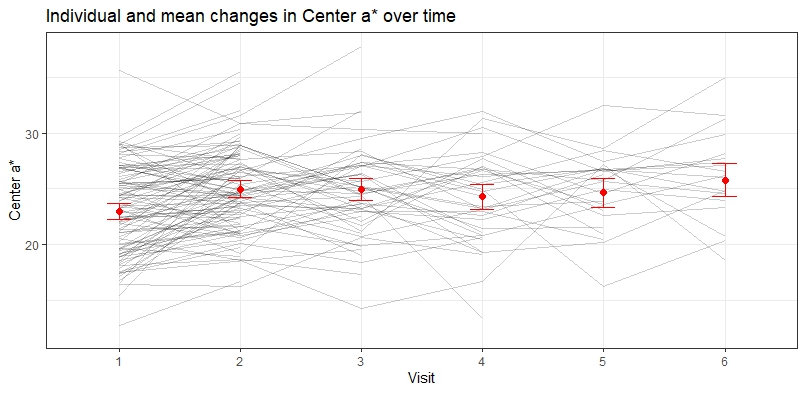 | 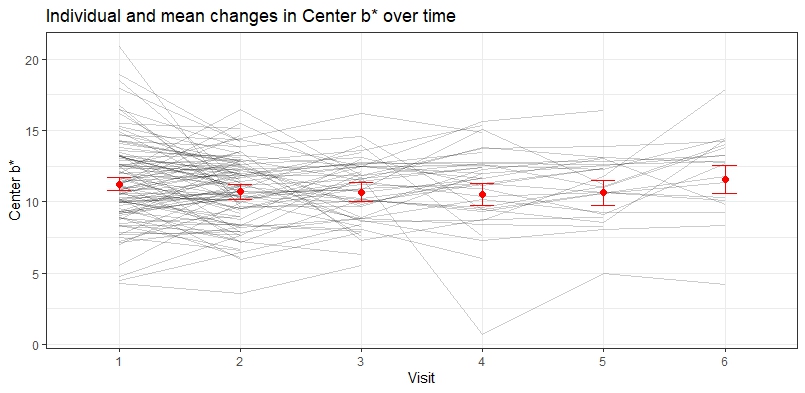 |
| 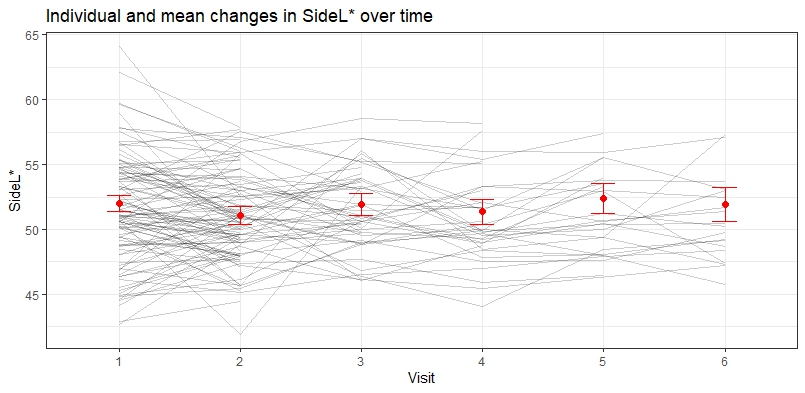 | 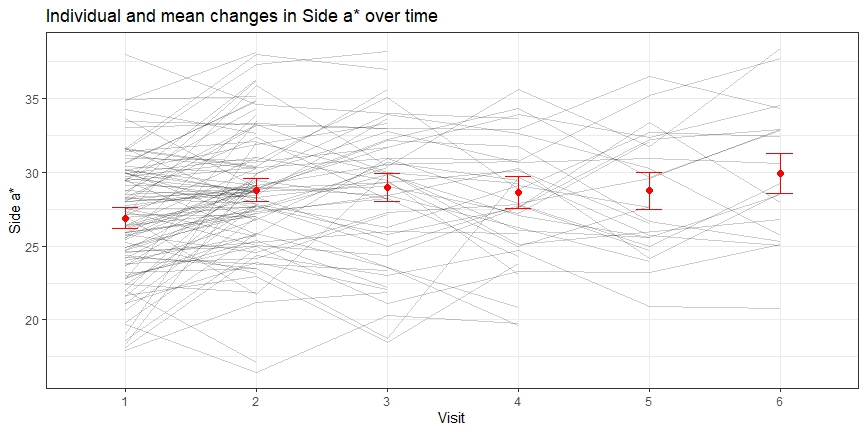 |
| 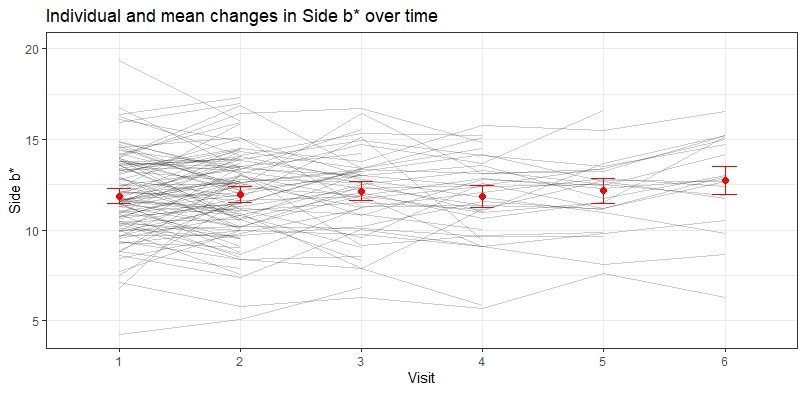 | 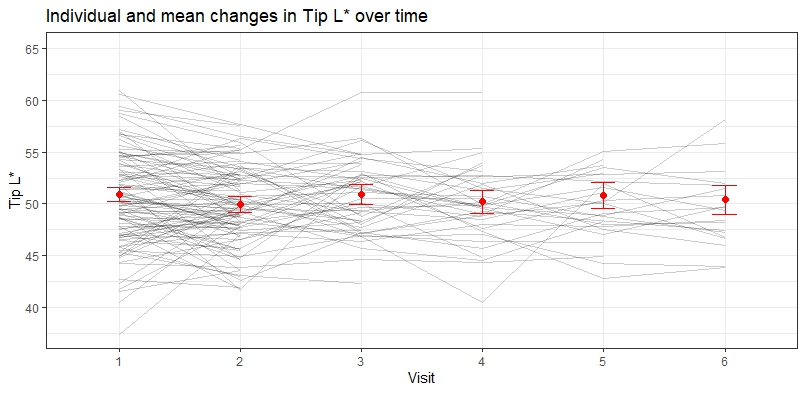 |
| 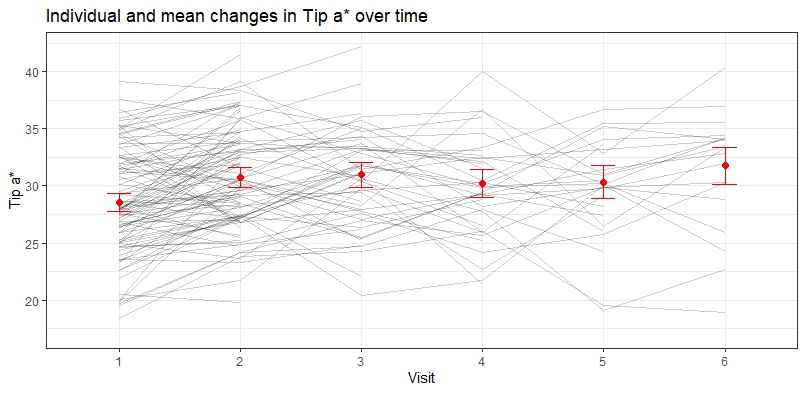 | 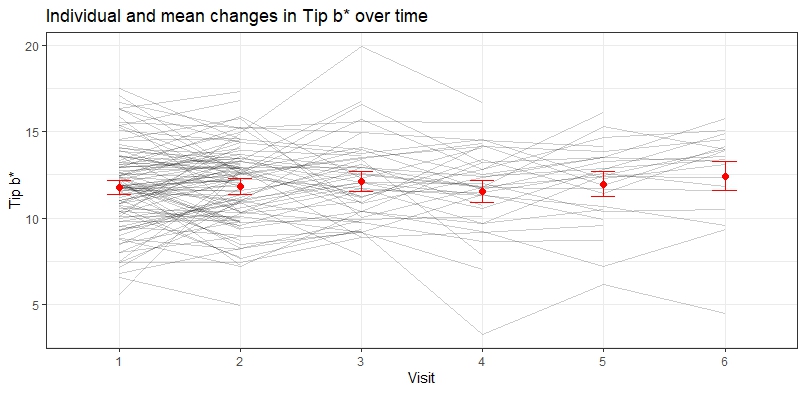 |
| 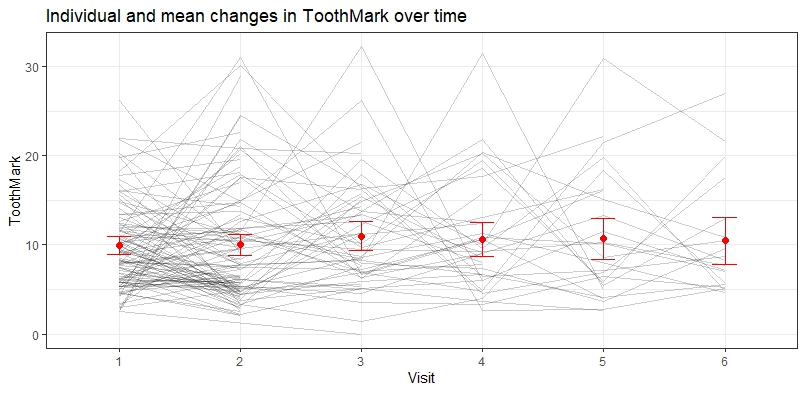 |  |
