# Supplementary material for: Temporal changes in tongue color during immune checkpoint inhibitor therapy in patients with non-small-cell lung cancer: a prospective observational study using digital tongue diagnosis
Source: Oncol Rev. 2025 Dec 9;19:1697252. doi: 10.3389/or.2025.1697252 (PMC12722973; doi:10.3389/or.2025.1697252)
Supplement: Supplementary file 4 [file Supplementaryfile4.docx]

Additional file 4. Kaplan–Meier survival curves for progression-free survival (PFS) and overall survival (OS) according to changes (Δ) in tongue lightness parameters

| **Model 1** (adjusting for sex and age) | |
| --- | --- |
| 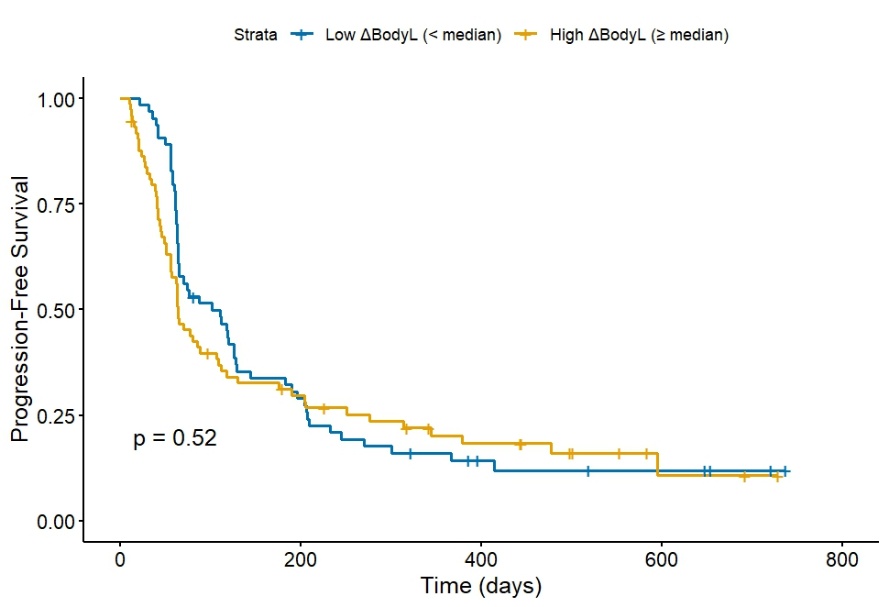 | 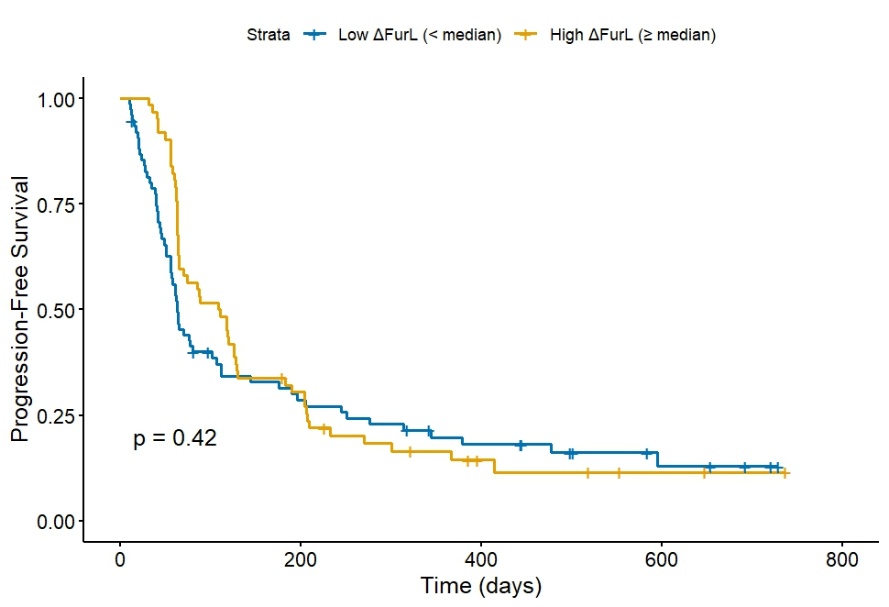 |
| PFS according to ΔBodyL | PFS according to ΔFurL |
| 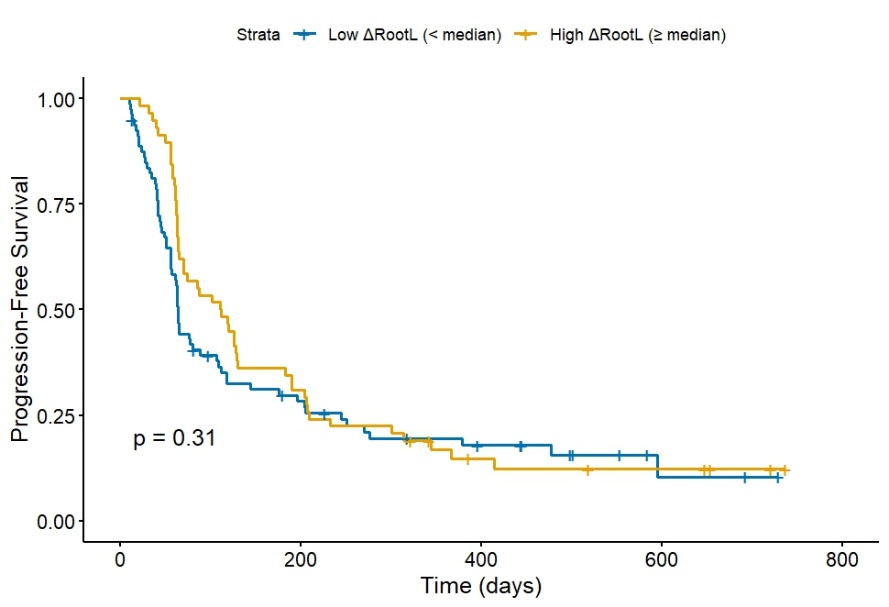 | 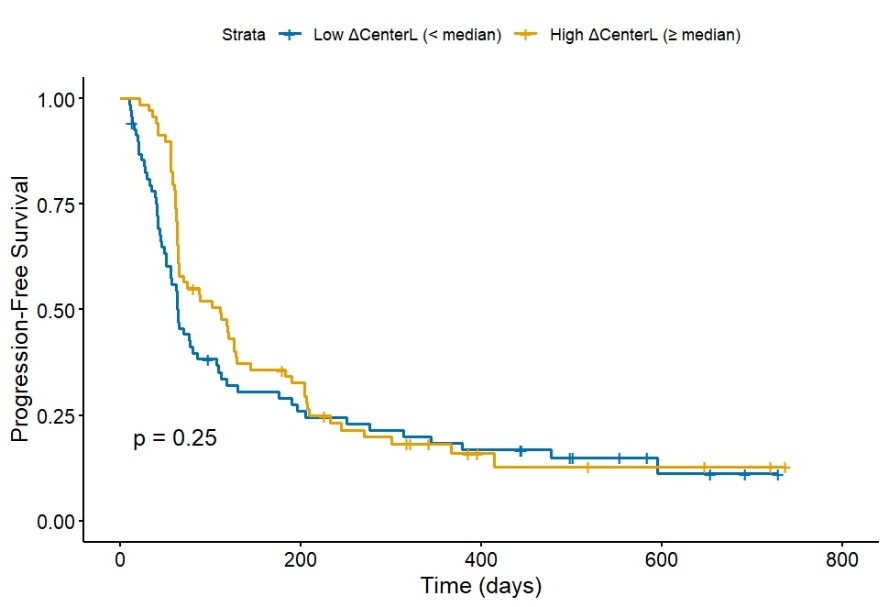 |
| PFS according to ΔRootL | PFS according to ΔCenterL |
| 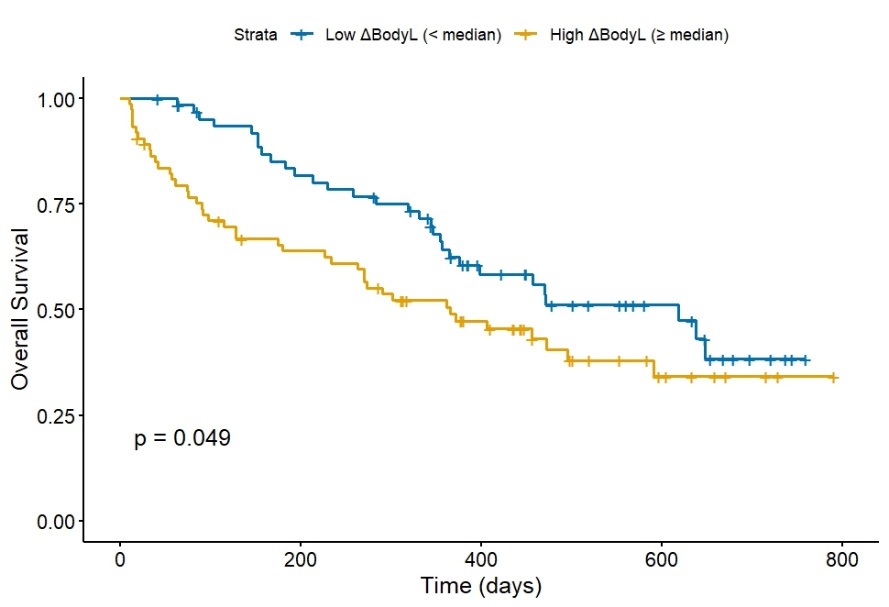 | 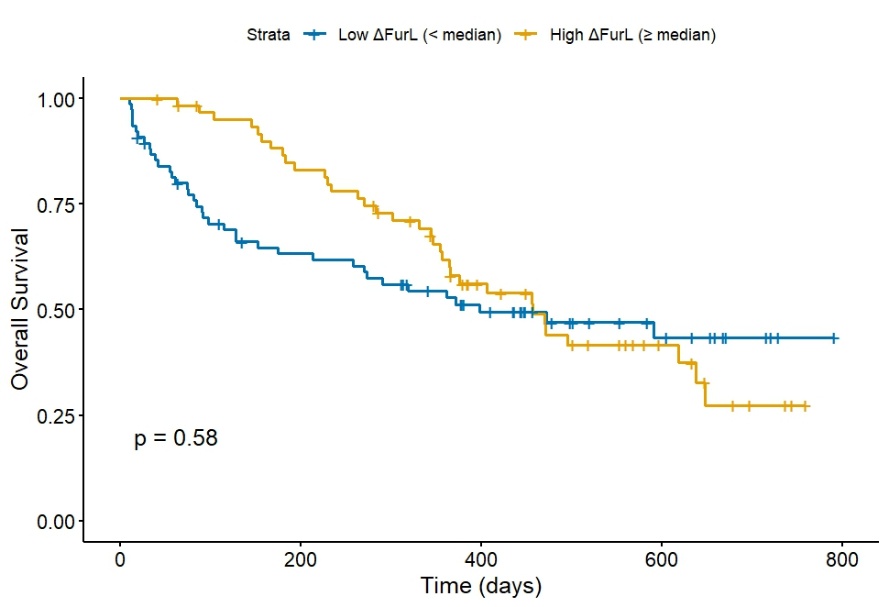 |
| OS according to ΔBodyL | OS according to ΔFurL |
| 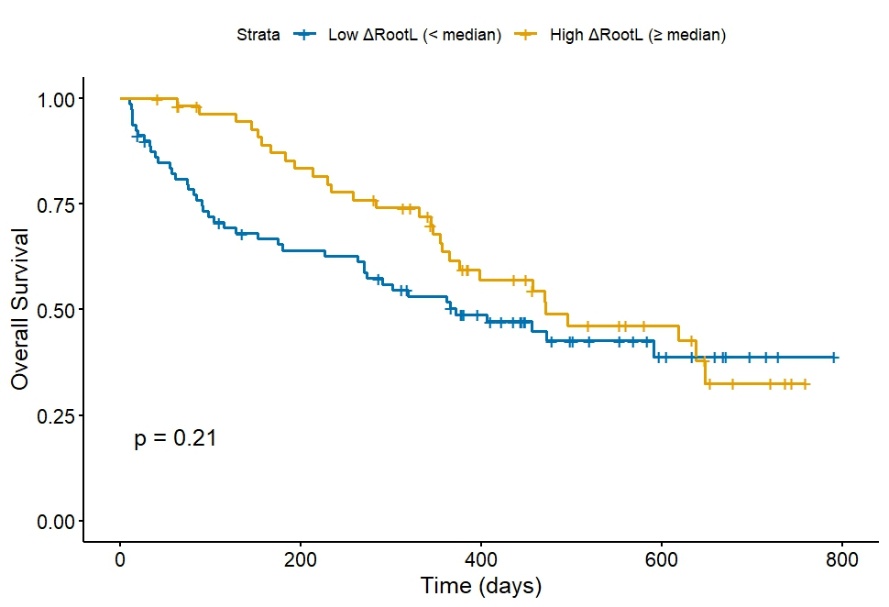 | 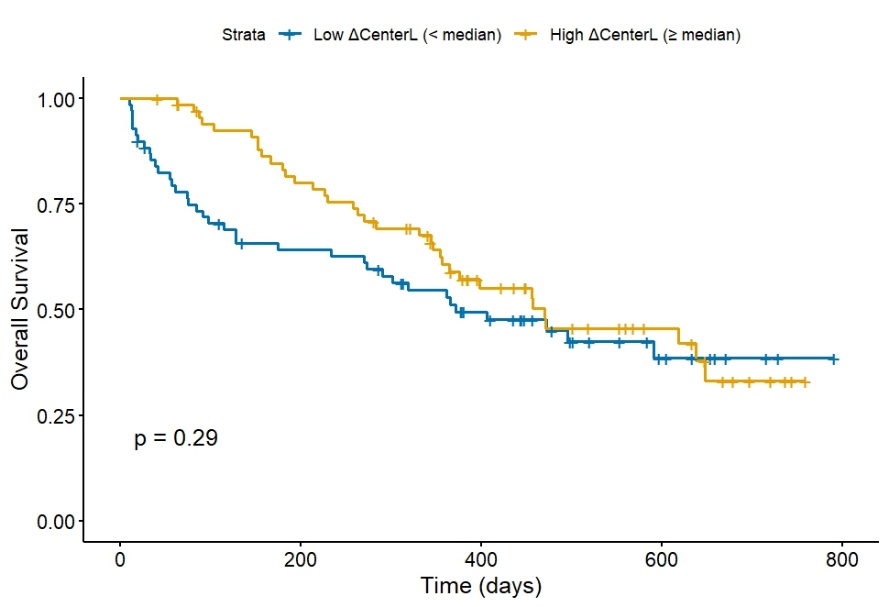 |
| OS according to ΔRootL | OS according to ΔCenterL |
|  |  |
| **Model 2** (adjusting for sex, age, PD-L1 expression, and ICI type) | |
| 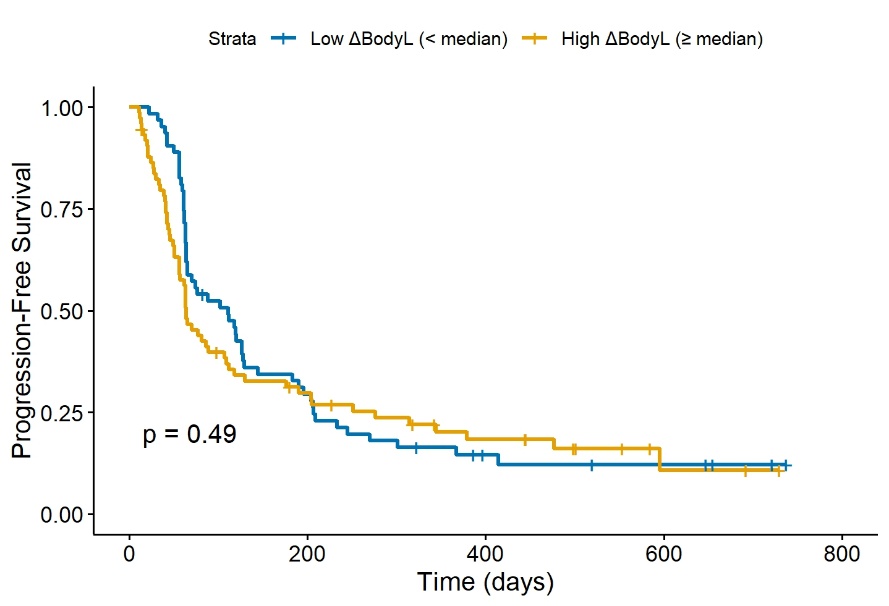 | 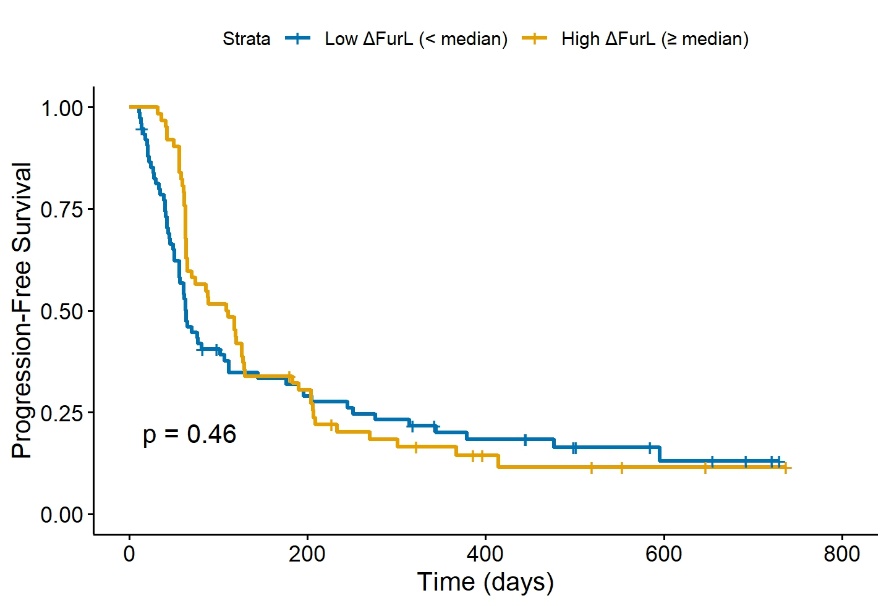 |
| PFS according to ΔBodyL | PFS according to ΔFurL |
| 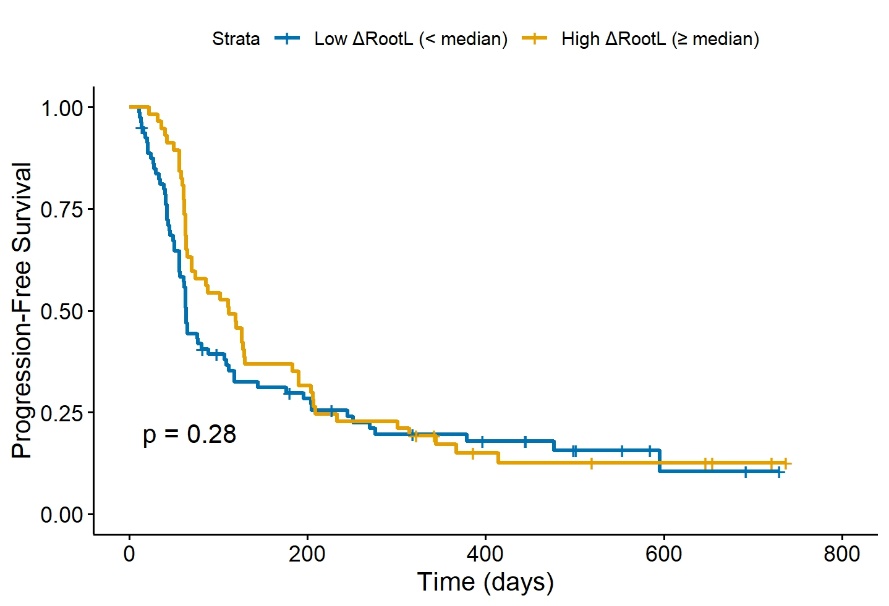 | 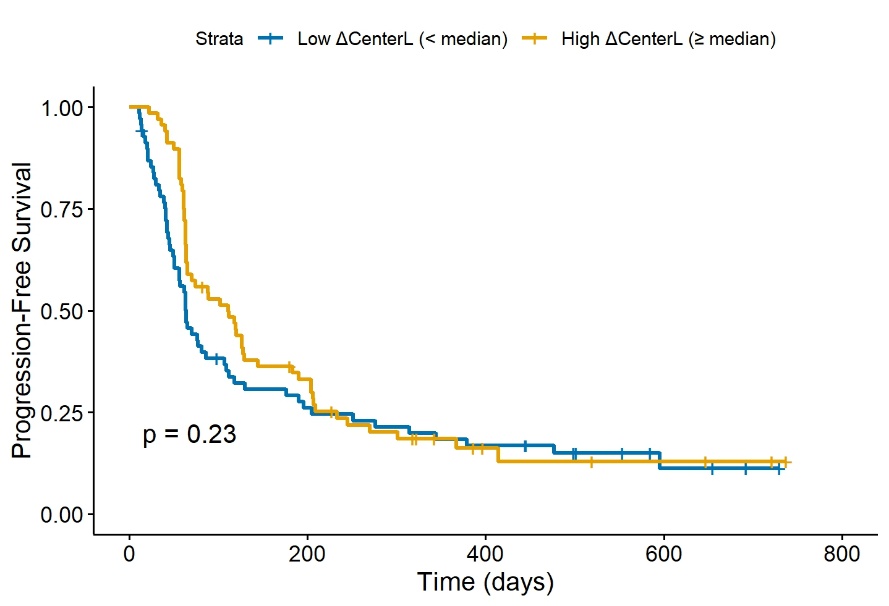 |
| PFS according to ΔRootL | PFS according to ΔCenterL |
| 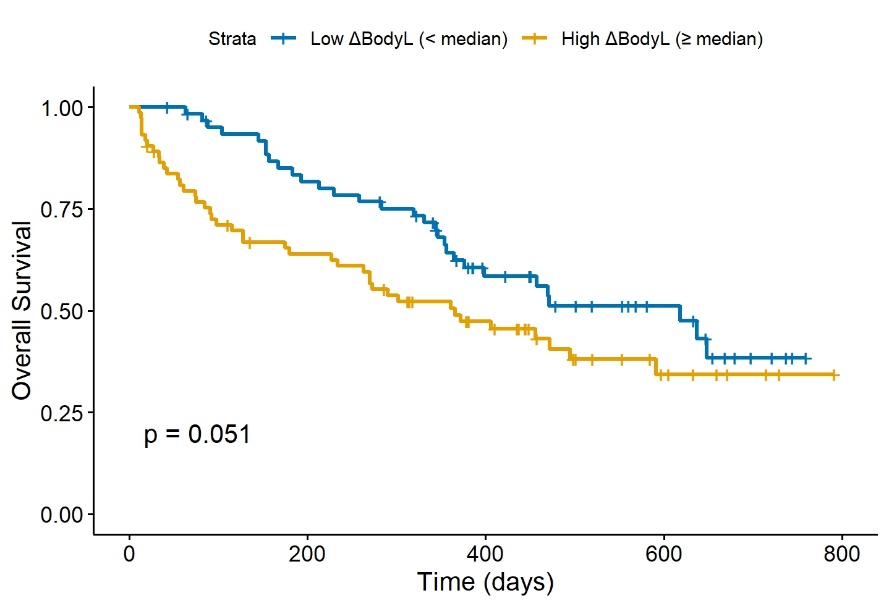 | 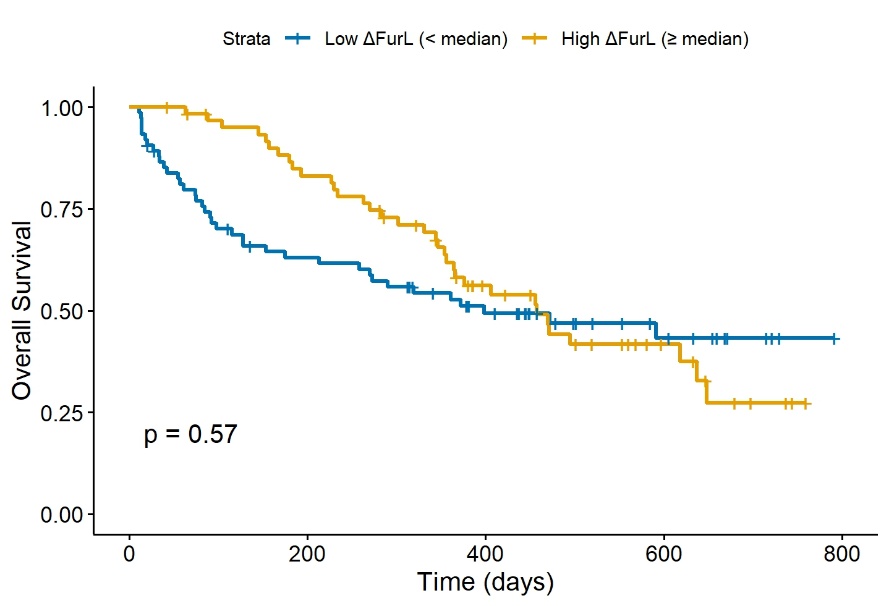 |
| OS according to ΔBodyL | OS according to ΔFurL |
| 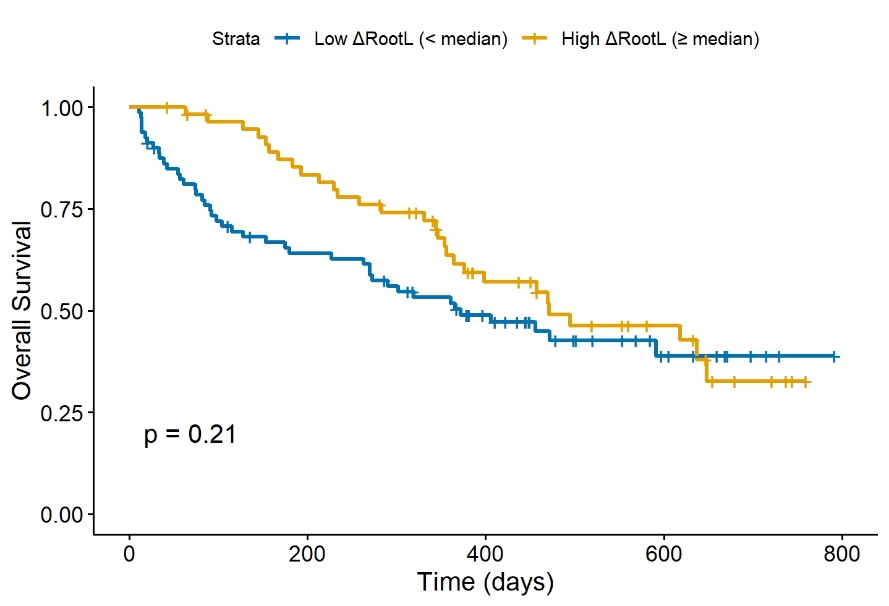 | 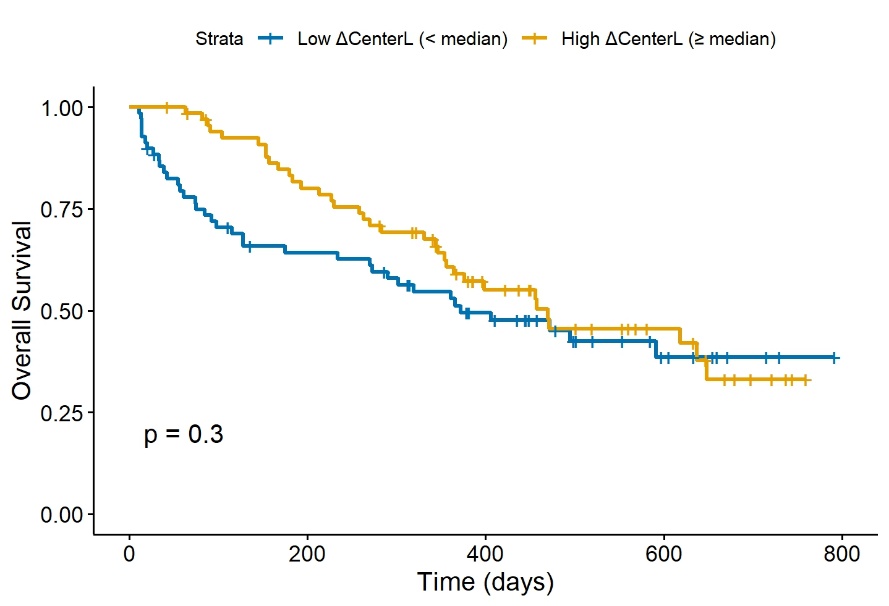 |
| OS according to ΔRootL | OS according to ΔCenterL |
